# Interpretable deep neural network identifies robust biomarkers for diseases with mechanistic insights from omics data

**DOI:** 10.1093/bib/bbag476

**Published:** 2026-09-07

**Authors:** Xiaoyue Hu, Yuhao Ma, Ruixing Ming, Heping Zhang, Hangjin Jiang

**Affiliations:** Center for Data Science, Zhejiang University, 866 Yuhangtang Road, Xihu District, Hangzhou, Zhejiang 310058, China; School of Mathematical Sciences, Zhejiang University, 866 Yuhangtang Road, Xihu District, Hangzhou, Zhejiang 310058, China; School of Statistics and Mathematics, Zhejiang Gongshang University, 18 Xuezheng Street, Xiasha Higher Education Zone, Hangzhou, Zhejiang 310018, China; School of Statistics and Mathematics, Zhejiang Gongshang University, 18 Xuezheng Street, Xiasha Higher Education Zone, Hangzhou, Zhejiang 310018, China; Department of Biostatistics, Yale University, 60 College Street, New Haven, CT, USA; State Key Laboratory for Vegetation Structure, Function and Construction (VegLab), Center for Data Science, Zhejiang University, 866 Yuhangtang Road, Xihu District, Hangzhou, Zhejiang 310058, China

**Keywords:** biomarker discovery, feature selection, artificial intelligence, deep neural networks

## Abstract

Identifying essential biomarkers remains a core challenge in elucidating the pathogenic mechanisms and achieving precise diagnosis of complex diseases. Deep neural networks offer immense predictive power, yet their lack of interpretability severely limits downstream biological insight. Here, we introduce DeepVaris, an explainable deep learning framework that reframes feature selection as the interpretation of a pretrained convolutional neural network via surrogate modeling. In extensive simulations and real-world datasets, DeepVaris successfully identifies important features and reveals deeper insight into different diseases. Specifically, it overcomes extreme feature sparsity to identify crucial microbial biomarkers in preterm birth pregnancies. In single-cell RNA sequencing data, it reveals key transcriptional drivers governing myelin regeneration in neurodegenerative diseases missed by traditional differential expression analysis. Furthermore, in complex breast cancer cohorts, DeepVaris moves beyond generic pan-cancer signals to precise subtype-specific microenvironmental targets. In summary, we believe that DeepVaris will serve as a robust tool for biomarker discovery.

## Introduction

The rapid advancement of biological technologies has driven the widespread application of feature (variable) selection across various fields, including target identification [1, 2], protein annotation [3], dimensionality reduction [4, 5], clinical phenotype prediction [6, 7], and disease-state characterization [8, 9]. Model-based methods, such as Lasso [10], Elastic Net [11], and Random Forests [12, 13], are commonly employed to solve feature selection problems, by evaluating the importance of each variable based on its contribution to the model performance. Recently, Stabl [7] has been proposed to adaptively choose important features by controlling the false discovery rate (FDR), which alleviates the selection of tuning parameters. Although these methods are interpretable, their effectiveness inherently relies on the underlying model assumptions, limiting their applicability to scenarios with unknown complex relationships between variables and outcomes. Additionally, they struggle with ultra-high-dimensional sequencing datasets due to their computational inefficiency in face of the sheer number of features and the low signal-to-noise ratios.

**Figure 1:**
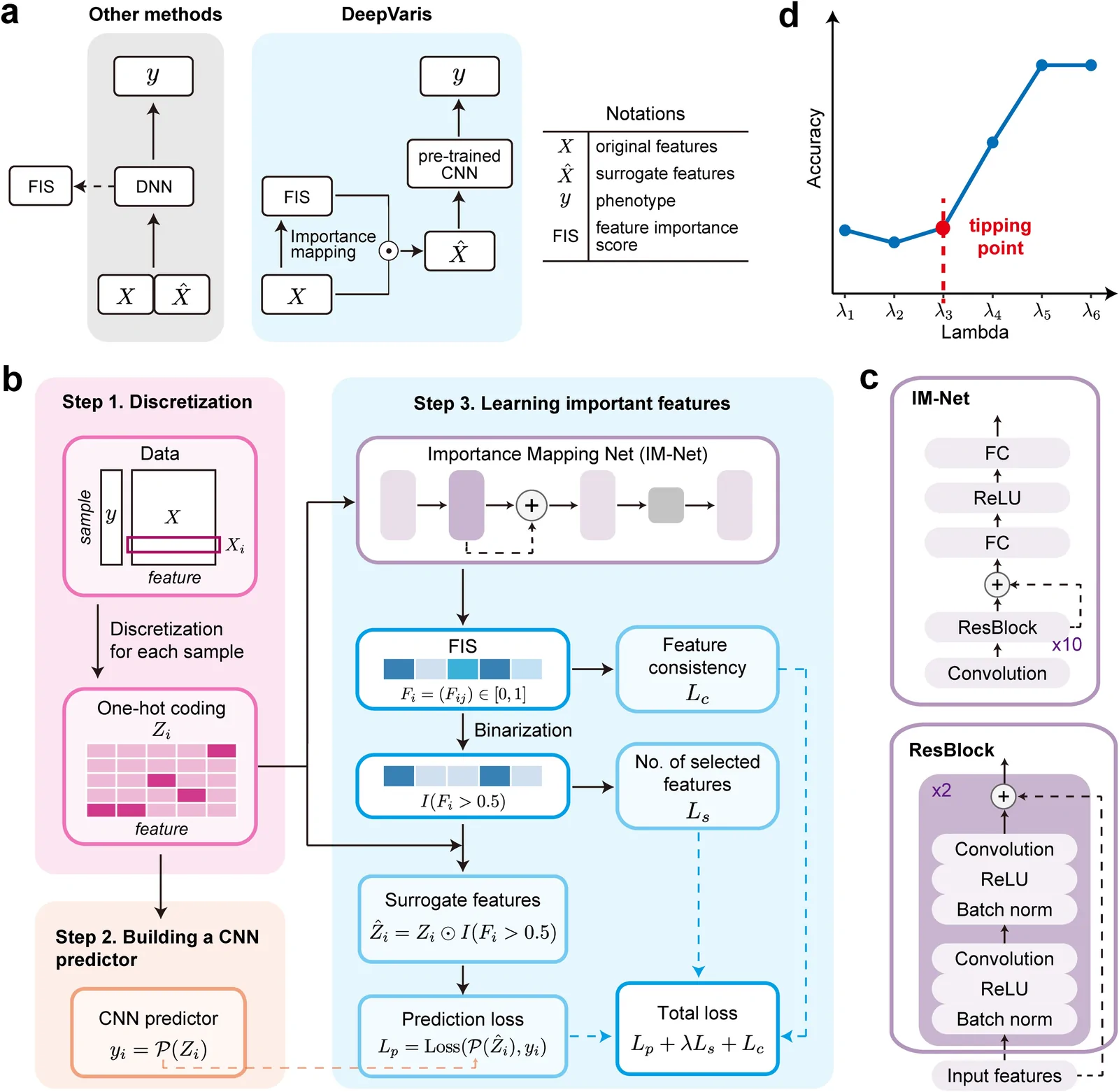
Overview of DeepVaris. **a**, Conceptual summary on ideas underlying DeepVaris and other methods to learn and define FIS. **b**, The architecture of DeepVaris. First, the input *X* is discretized and transformed into an image *Z*. Second, taking $D=\{(Z_{i}, y_{i}):i=1,2\cdots , n\}$ as input, a CNN predictor $\mathcal{P}$ is trained to predict the outcome *y*. Third, taking *D* and $\mathcal{P}$ as input, an Importance Mapping Net (IM-Net) is trained to learn feature importance by minimizing the prediction error of the pretrained CNN ($\mathcal{P}$) using surrogate features ($\hat{Z}_{i}$) with constraints on the number of features ($L_{s}$, **Methods**) and the feature consistency ($L_{c}$, **Methods**). **c**, The architecture of IM-Net. **d**, The parameter *λ* in total loss of DeepVaris is chosen as the tipping point in the performance of pretrained CNN model, evaluated on the remaining features after removing important ones.

Deep neural networks (DNNs) can efficiently capture the complex relationships in data, including both linear and nonlinear effects, through their hidden layers without explicit specification [14]. In light of this, several DNN-based feature selection methods, such as SurvNet [15], DeepLink [16], HiDe-MK [17], have been proposed and gained popularity for their enhanced performance. In principle, they follow the same idea to identify important variables by comparing feature importance score (FIS) of original variables with that of knockoff/surrogate variables after the DNN is trained (Fig. 1a). Here, the key element is FIS, and knockoff/surrogate variables are used to control the FDR. Specifically, SurvNet defines FIS as the mean absolute gradient of the loss function in the trained DNN, while HiDe-MK uses the difference between the output gradients of original and knockoff variables. DeepLink derives FIS from the weights of pairwise-connected filter layer together with the downstream multilayer perceptron (MLP) weights.

Despite their utility, these methods face three major challenges. First, surrogate or knockoff variables introduce artificial noise. They double the feature dimension from *p* to *2p*. This greatly complicates model training, particularly in datasets where features far outnumber samples (*p ≫ n*). Second, selecting an appropriate FDR threshold is highly subjective in practice. A strict cutoff often forces the exclusion of critical factors. In high-throughput data with weak signals, this severe sparsity distorts the true biological mechanisms. Third, FIS heavily relies on the model’s goodness-of-fit. An overfitted or underfitted network inevitably produces biased scores and false positives. In other words, DNNs with identical predictive accuracy can select fundamentally different feature sets. This inconsistency poses a severe risk of misleading downstream analysis.

To address these challenges, we propose DeepVaris, an interpretable DNN-based method that puts feature selection into the framework of interpreting a convolutional neural network (CNN) predictor through surrogate modeling. Rather than deriving feature importance directly from model parameters, DeepVaris learns a sparse feature mask that preserves the predictive behavior of the CNN, thereby identifying variables that contribute to its decisions. By leveraging the ability of CNNs to capture complex nonlinear patterns, DeepVaris can recover conditionally informative features beyond marginal associations. A batch-consistency constraint further favors stable selections and reduces random false discoveries. Feature-set complexity is regulated by an $L_{0}$ penalty, and the corresponding regularization parameter is selected according to the predictive performance of the unselected variables, without requiring a prespecified FDR threshold. Benchmarking on extensive simulations and three real-world datasets demonstrates the substantial advantages of DeepVaris over the state-of-the-art methods.

## Results

### Overview of DeepVaris

Notably, CNNs excel at automatically extracting important features from images and demonstrate robustness to noisy data (Fig. S1). DeepVaris leverages this strength, and identifies important features by interpreting what a pretrained CNN has already learned. Conceptually, we introduce an Importance Mapping Net to calculate the FIS for each variable. Specifically, the workflow proceeds as follows (Fig. 1b and c; **Methods**). First, we discretize continuous variables to convert tabular input into an image format. We then train a CNN to accurately predict the target outcome. Next, we apply surrogate modeling to interpret this network. The Importance Mapping Net assigns an FIS (*∈ [0,1]*) to each feature, constrained by sparsity and batch consistency. Features scoring above 0.5 are selected as surrogate features. To validate their quality, we feed only these surrogates back into the pretrained CNN. If the selected features capture the true biological drivers, the CNN’s predictive performance will remain intact.

This principle fundamentally distinguishes DeepVaris from existing approaches. Conventional methods derive FIS directly from the trained DNN, and an improperly trained model inevitably produces biased scores. DeepVaris avoids this trap based on three new points (Fig. 1a). (i) A new perspective on feature selection. We reframe biomarker discovery as interpreting a pretrained CNN by surrogate modeling. (ii) Empirically low FDR via feature stability. Noting that false positives are random and batch-specific, whereas true features are stable across samples, we reduce the false positives by enforcing batch consistency and convergence across multiple trains for FIS learning (**Methods**). (iii) Dynamic parameter tuning. The tuning parameter *λ* for regulating the model complexity (the number of features) is selected as the tipping point of the performance of pretrained CNN model taking unselected features as input, i.e. the features discarded by DeepVaris (Fig. 1d, **Methods**). In summary, DeepVaris captures complex nonlinear relationships while inherently guarding against false discoveries.

### DeepVaris outperforms its competitors in simulations

DeepVaris is comprehensively benchmarked against state-of-the-art feature selection methods, including Stabl [7], SurvNet [15], DeepLink [16], and HiDe-MK [17], across six simulation examples covering both classification (Datasets 1–3) and regression (Datasets 4–6) tasks with sample size *n=10 000*, number of features *p=784*, and number of true features *s=64* (**Methods**). The FDR of SurvNet, DeepLink, and HiDe-MK are controlled at 10%. Performance is evaluated using prediction error (final error), FDR, and the ratio of selected true features to all true features (TR), with a lower final error and FDR, and a higher TR indicating better performance. Results are reported from 50 independent repetitions.

**Figure 2:**
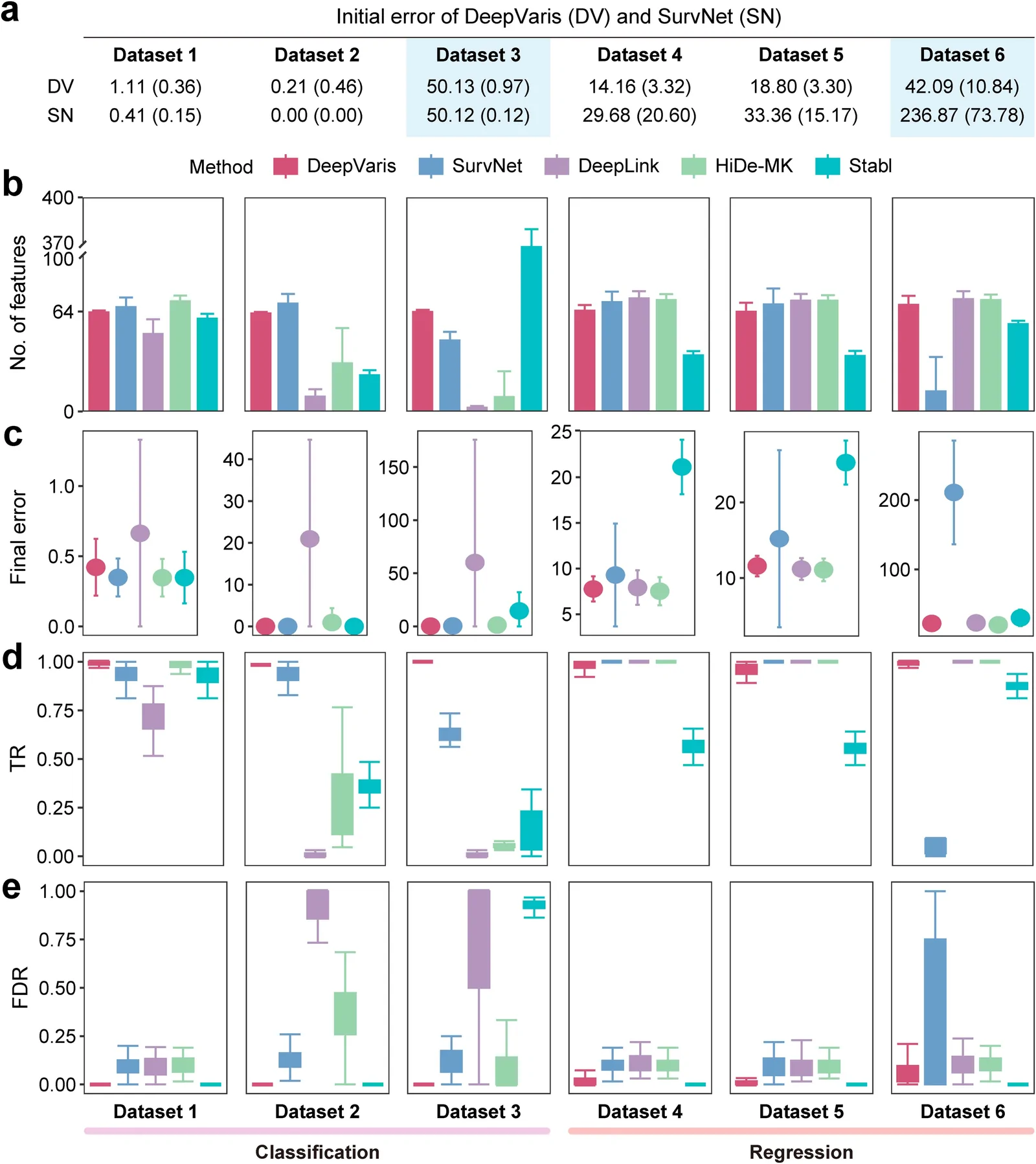
DeepVaris outperforms competing methods in simulations. **a**, The initial error of DeepVaris and SurvNet on six simulated datasets, each with a sample size of *n=10 000* and *p=784* variables. **b**, The number of features selected by each method. Note that the number of true variables is *s=64*. **c–e**, The performance of different methods on six simulated datasets measured by **c**, final test error, **d**, TR (the ratio of true features selected by each method, ranging from 0 to 1 and a larger TR indicates a better performance), and **e**, FDR.

DeepVaris demonstrates superior performance across all datasets, achieving TR >95%, FDR <7%, and comparably small final errors, while competing methods exhibit significantly poorer performance on certain datasets (Fig. 2b–e). Specifically, SurvNet performs poorly on Dataset 6, with a TR as low as 17%, while DeepLink, HiDe-MK, and Stabl show inferior performance on Datasets 2 and 3, with much lower TR and higher FDR (Fig. 2d and e). Notably, in Dataset 3 and 6, which are two noisy datasets with higher initial errors (Fig. 2a), SurvNet shows poor performance with higher FDR and lower TR, while DeepVaris significantly outperforms other competing methods (Fig. 2b–e). Importantly, a small test error, often regarded as an indicator of good classification or regression performance, does not necessarily indicate good performance on feature selection (Fig. 2c). For example, in Dataset 2 (a classification task), although Stabl controls the FDR very well and achieves a final test error of 0, it selects only on average 23 out of the 64 true features, resulting in a low TR of 36% (Fig. 2b–d). Similar phenomena is observed for HiDe-MK in this example. In Dataset 4 (a regression task), DeepLink attains a comparable test error with DeepVaris but selects a larger number of false variables, leading to a higher FDR (Fig. 2b–e). Thus, while some methods achieve lower test errors, they may overlook important features or include spurious ones, complicating downstream validation and analysis. In contrast, DeepVaris consistently outperforms other methods by selecting nearly all the 64 true features with low empirical FDR (Fig. 2b–e).

Given the best performance of DeepVaris, SurvNet, and DeepLink in these examples (Fig. 2), we further compare these three methods across diverse settings with different sample sizes (*n=1000, 5000, 10 000*) and numbers of features (*p=784, 2000, 4000, 6000, 10 000*) in four representative examples (Dataset 1, 3, 4, 6). Overall, DeepVaris exhibits robust performance with higher TR but lower FDR (Figs S2–S5). For instance, in Dataset 4, when the sample size is 5000, DeepVaris achieves average TR values of 0.98, 0.97, 0.95, 0.96, and 0.92 as *p* increases from 784 to 10 000 (Fig. S4). In contrast, the performance of SurvNet is strongly affected by *p*, with TR values of 0.96, 0.91, 0.65, 0.45, and 0.29 under the same settings (Fig. S4). Moreover, SurvNet exhibits instability, often selecting either many or none of the variables under different settings of *p* and *n* (Figs S4 and S5). Finally, DeepLink achieves comparable performance with DeepVaris in regression tasks (Figs S4 and S5), but performs notably inferior in classification tasks (Figs S2 and S3), limiting its applicability in real world scenarios.

In summary, DeepVaris is preferred for its efficacy, high true positives, versatility across classification and regression tasks, and robustness to noise. Comparatively, SurvNet requires models with small initial errors, otherwise its performance is undesired. DeepLink and HiDe-MK perform well in regression tasks but worse in classification tasks, consistent with previous studies [16, 17]. Different from other methods, DeepVaris automatically suppresses the empirical FDR at a low level without a predefined threshold, e.g. $\leqslant 10\%$, when sample size is large enough.

### DeepVaris rescues synergistic biomarkers for preterm birth prediction

**Figure 3:**
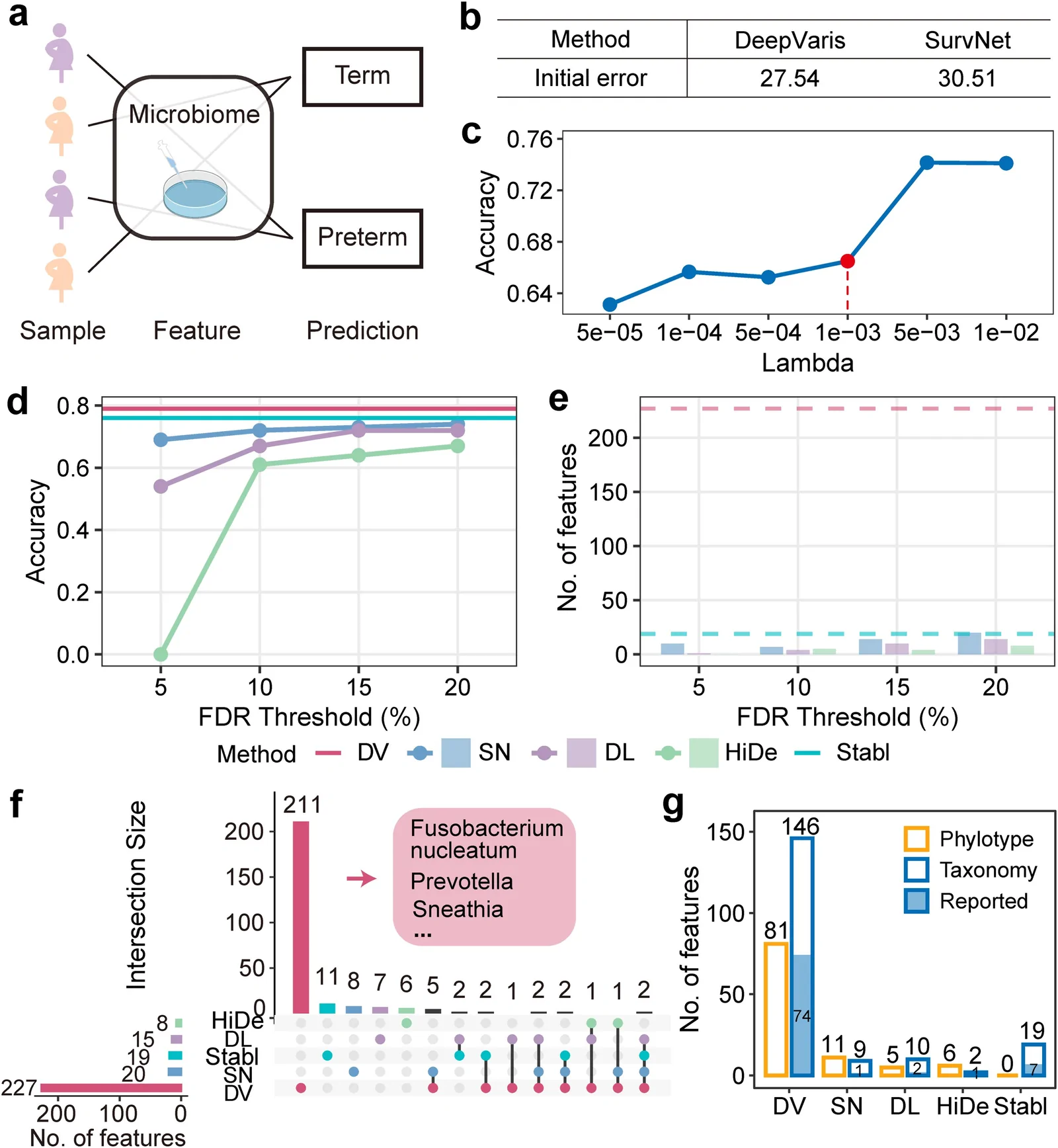
DeepVaris rescues synergistic biomarkers for preterm birth prediction. **a**, The description of Dream dataset. **b**, The initial errors of DeepVaris and SurvNet. **c**, The parameter *λ* in DeepVaris is chosen as the tipping point of test accuracy. **d** and **e**, The performance of five feature selection methods, DeepVaris (DV), SurvNet (SN), DeepLink (DL), HiDe-MK (HiDe), and Stabl, under different FDR thresholds, reported by **d**, prediction accuracy and **e**, number of selected features. Note that the selection of DV and Stabl are independent of the threshold. **f**, The relationship between the features selected by different methods with optimal predicted accuracy. Features in the box represent examples of reported features only identified by DeepVaris. **g**, The number of features from different types, i.e. phylotype and taxonomy, selected by different methods. The numbers inside the bars indicate the numbers of selected features reported in literature.

Dysbiosis of the host microbiome is intricately associated with the pathogenesis of systemic syndromes such as preterm birth. However, due to the inherent high dimensionality, low signal-to-noise ratio nature of microbiome abundance data, true but subtle signals are frequently eclipsed by overwhelming environmental background noise. Here, we focus on the Microbiome Preterm Birth DREAM challenge, which seeks to classify preterm and term labor pregnancies using microbiome datasets comprising phylotypic and taxonomic variables [7] (Fig. 3a; **Methods**). This task is particularly challenging, as evidenced by the substantial initial errors of DeepVaris and SurvNet (Fig. 3b). We apply DeepVaris (DV), alongside cutting-edge models like SurvNet (SN), DeepLink (DL), HiDe-MK (HiDe), and Stabl, to select important features.

To ensure a rigorous and fair evaluation, we assess the predictive performance across a spectrum of sparsity levels. We systematically relax the FDR thresholds for competing methods (ranging from 5% to 20%) to encourage broader feature selection. Notably, the selection of DeepVaris and Stabl are independent of predefined thresholds. DeepVaris intrinsically determines the optimal feature sets via a data-driven signal depletion strategy, identifying *λ* as the tipping point of test accuracy evaluated on un-selected features (Fig. 3c), while Stabl automatically minimizes the false discovery proportion within a specified range. Overall, DeepVaris consistently outperforms all other methods in terms of predictive accuracy (Fig. 3d). Strikingly, even when we relax the FDR thresholds of competing methods up to 20%, they still select fewer than 20 variables and yield sub-optimal accuracies (Fig. 3d and e). In contrast, DeepVaris identifies a much broader set of over 200 variables. This disparity highlights a severe false-negative penalty inherent in rigid FDR-based methods. By leveraging the signal depletion strategy, DeepVaris successfully captures weak signals that lack individual marginal strength but collectively form a highly predictive, synergistic network.

To confirm that this expanded feature set is biologically relevant rather than high false positives, we further compare the feature sets that yield the optimal predictive performance for each approach (Fig. 3f). While phylotypic variables lack supporting references, taxonomic variables can be validated by existing literature. Among the selected taxonomic features, competing methods only manage to recover single-digit relevant variables (Fig. 3g). Conversely, DeepVaris successfully rescues 74 previously reported, preterm-associated features, achieving a remarkable validation proportion of over 50% (Fig. 3f and g; Supplementary Data I). These recovered features actively affect preterm birth through diverse mechanisms, such as promoting inflammatory response (e.g. Fusobacterium nucleatum [18], Prevotella [19]), influencing immune system (e.g. Lactobacillus [20], Bifidobacterium [21]), or interacting with other pathogens (e.g. Sneathia [22], Peptostreptococcaceae [23]). For instance, Fusobacterium nucleatum infection is accompanied by inflammation responses and associated with an increased risk of preterm birth [18], while Prevotella spp. and Genus Sneathia have been reported to be more prevalent in preterm infants [19, 22]. Thus, while methods depending on predefined FDR threshold suffer from losing important features, DeepVaris provides a substantially richer and highly validated pool of candidates for downstream analysis.

### DeepVaris captures key regulators of remyelination in neurodegenerative diseases

**Figure 4:**
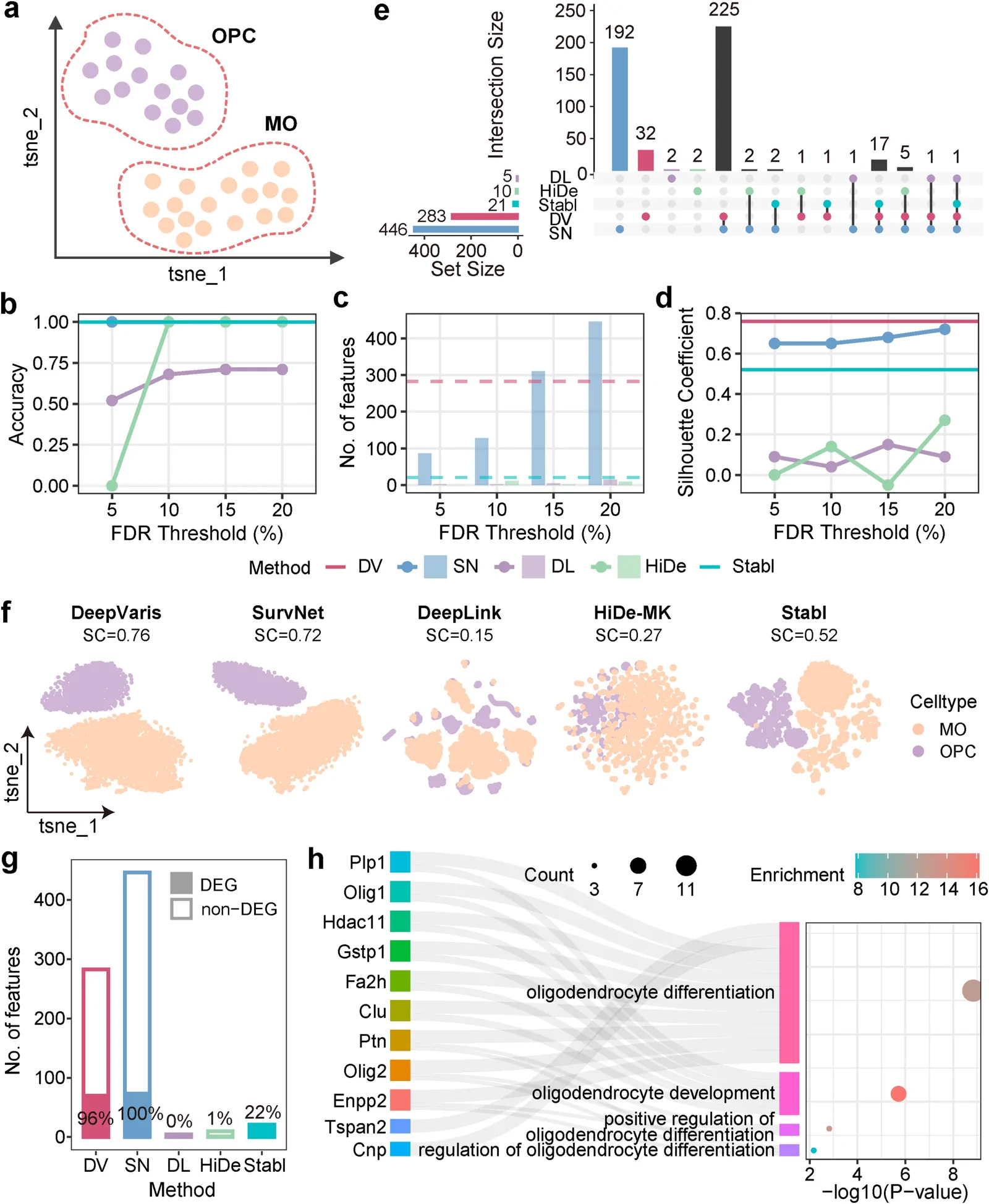
DeepVaris captures key regulators of remyelination in neurodegenerative diseases. **a**, The description of scRNA mouse dataset. **b–d**, The performance of five feature selection methods, DeepVaris (DV), SurvNet (SN), DeepLink (DL), HiDe-MK (HiDe), and Stabl, under different FDR thresholds, reported by **b**, prediction accuracy, **c**, selected number, and **d**, clustering quality measured by the SC. Higher SC values indicate superior cell-type clustering results. Note that the selection of DV and Stabl are independent of the threshold. **e**, Overlap of the feature sets that yield the optimal clustering result for each method. **f**, The t-SNE plots showing the optimal clustering result. **g**, Composition of the selected features, categorized into standard DEGs and non-DEGs. Percentages denote the proportion of the 73 DEGs successfully recovered by each method. **h**, The pathways related to oligodendrocyte maturation, enriched by non-DEGs selected by DeepVaris. In the left, we show examples of these genes.

We next apply DeepVaris at single-cell resolution to investigate key drivers governing myelin regeneration in neurodegenerative diseases. Since impaired remyelination is a major barrier to treating conditions like multiple sclerosis (MS) [24], identifying the transcriptional switches that drive oligodendrocyte precursor cells (OPCs) to mature into myelinating oligodendrocytes (MOs) is of critical clinical importance. Therefore, we analyze an adult mouse hypothalamus dataset representing these two distinct developmental stages [15] (Fig. 4a). Again, we compare DeepVaris against other four methods, with their FDR thresholds ranging from 5% to 20%.

Overall, this classification task is relatively straightforward, as both DeepVaris and SurvNet exhibit initial errors of 0, and most methods (except DeepLink) achieve perfect predictive accuracy (Fig. 4b, Fig. S6a). However, consistent with our simulations, methods yielding similar test accuracies produce vastly different feature sets (Fig. 2). While DeepLink, HiDe-MK, and Stabl impose severe sparsity (fewer than 25 features), SurvNet selects a highly expanded set (100–400 features) as its FDR threshold relaxes from 5% to 20%. DeepVaris identifies an intermediate set of 283 features (Fig. 4c, Fig. S6b).

To determine which feature set best reflects the underlying biological manifold, we perform t-Distributed Stochastic Neighbor Embedding (t-SNE) dimensionality reduction based on selected features and quantify the cell-type separation using Silhouette Coefficient (SC) metric (**Methods**). DeepVaris consistently outperforms all competitors, achieving the highest SC of 0.76 (Fig. 4d–f). In contrast, the overly sparse feature sets of DeepLink and HiDe-MK fail to adequately separate the cell types (SC *⩽* 0.3), indicating a critical loss of biological signal. Conversely, SurvNet yields suboptimal clustering despite its massive feature size, revealing a penalty from feature inflation and noise incorporation (Fig. 4d). DeepVaris, therefore, optimally balances sparsity with signal recovery.

Subsequent pathway enrichment analysis reinforces this balance. Based on the features yielding the best clustering result for each method (Fig. 4e), DeepVaris enriches six pathways closely linked to oligodendrocyte maturation (Fig. S6c). Notably, two of these core pathways are exclusively identified by DeepVaris, confirming its superior sensitivity to biological transitions.

Finally, we cross-reference these signatures against standard differentially expressed genes (DEGs). Both DeepVaris and SurvNet successfully recover the vast majority of DEGs, in contrast to the limited recovery rates observed in the highly sparse methods (Fig. 4g). To evaluate the advantage of DeepVaris over DEG, we examine the non-DEG subset, which are the features exclusively rescued by our model. Biologically, this subset contains previously validated drivers of oligodendrocyte differentiation, including Olig1/2 [25], Plp1 [26], Fa2h [27], Clu [28], Ptn [29], Enpp2 [30], Tspan2 [31], and Cnp [32]. Specifically, Olig1 and Olig2 are expressed exclusively by oligodendrocytes after late embryonic development [25], while Plp1 and Cnp are highly expressed during oligodendrocyte maturation and involved in myelination [26, 32]. Their importance is further supported by the enrichment in pathways associated with oligodendrocyte maturation (Fig. 4h, Supplementary Data II). Theoretically, the exclusion of these crucial genes by DEG analysis (DEGA) highlights a fundamental statistical limitation. DEGA evaluates the isolated marginal effects of genes, inherently neglecting variables with weak marginal but strong conditional effects. DeepVaris, as a model-based framework, captures these conditional synergies. Interestingly, while SurvNet also enriches similar synergistic developmental pathways from its non-DEG subset, it requires an inflated repertoire (*⩾*400 variables) to capture this hidden network (Fig. S6d). DeepVaris delineates the exact same synergistic mechanisms using a highly refined, compact module (Fig. 4h). Collectively, these results demonstrate that DeepVaris provides a more precise, robust, and biologically comprehensive feature representation.

### DeepVaris isolates subtype-specific drivers in breast cancer treatment

**Figure 5:**
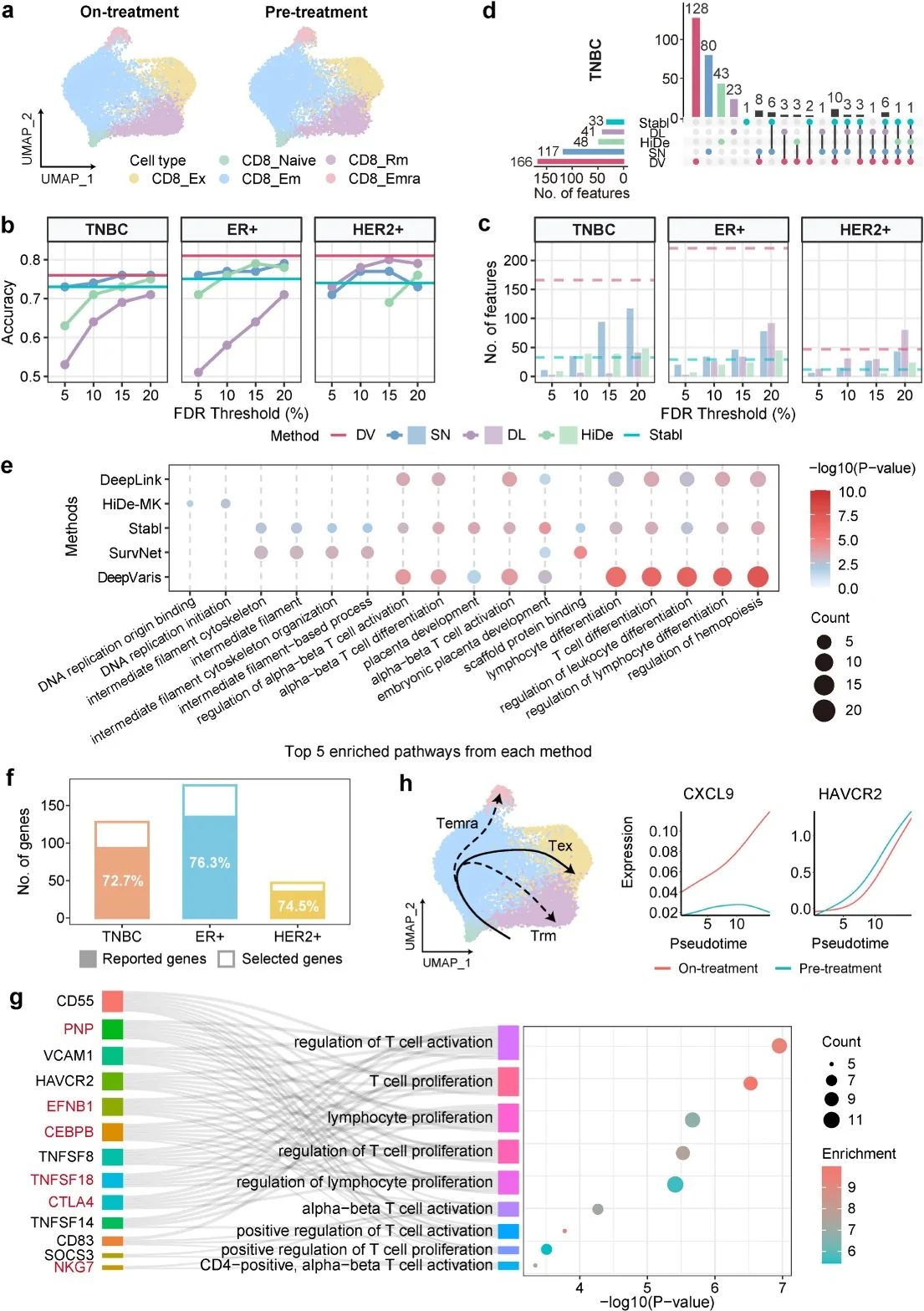
DeepVaris isolates subtype-specific drivers in breast cancer. **a**, The description of BC dataset. **b** and **c**, The performance of five feature selection methods, DeepVaris (DV), SurvNet (SN), DeepLink (DL), HiDe-MK (HiDe), and Stabl, in each subtype of BC under different FDR thresholds, reported by **b**, prediction accuracy and **c**, selected number. Note that the selection of DV and Stabl are independent of the threshold. **d**, The relationship between the optimal gene sets selected by different methods in TNBC data. **e**, The top five most significantly enriched pathways derived from the optimal feature sets selected by DeepVaris and competing methods within the ER+ BC cohort. **f**, Bar plot showing the number of genes exclusively identified by DV, and the proportion of genes reported in literature to be correlated with BC treatment among them. **g**, The pathway enrichment of genes selected by DV but ignored by other methods (in the left, we show examples of these genes, where genes in red are non-DEGs). These pathways are all related to the treatment of TNBC. **h**, Trajectory analysis along the Tex trajectory in TNBC data on examples of important markers only identified by DV, where CXCL9 is a marker of immune activation and HAVCR2 is an important marker for immune exhaustion.

Finally, DeepVaris excels at decoding transcriptomic dynamics during therapy, pinpointing key immune-exhaustion biomarkers associated with treatment response. Here, we apply DeepVaris to CD8+ T cells from breast cancer (BC) patients before (pre-) and during (on-) anti-PD1 immunotherapy [33] to identify critical genes and synergistic pathways driving treatment response (Fig. 5a). This dataset contains three BC subtypes: triple-negative (TN), ER-positive (ER+), and HER2-positive (HER2+) BC. We develop classification models for each subtype, and all of them pose significant challenges, as evidenced by the large initial errors of DeepVaris and SurvNet (Fig. S7a).

Consistent with previous findings, DeepVaris maintains its robust predictive advantage across all three subtypes, whereas the inferior results of competing methods highlight that stringent FDR thresholds (e.g. 5%) severely reduce predictive power (Fig. 5b). Accordingly, we investigate the features that yield the optimal prediction results for each method (Figs 5c and d, S7c). To evaluate how these selected features translate into biological mechanisms, we compare their top five enriched pathways from each method (Figs 5e, S8a and b). As expected, hindered by severe feature sparsity, HiDe-MK, DeepLink, and Stabl may fail to yield any significant networks (e.g. the HER2+ cohort in Fig. S8b) or predominantly capture generic pan-cancer hallmarks of proliferation, such as DNA replication (Figs 5e, S8a). In contrast, methods with larger feature sets demonstrate superior explanatory depth. SurvNet successfully recovers BC-associated microenvironmental signatures, capturing intermediate filament organization and broad myeloid cell differentiation (Figs 5e, S8b). DeepVaris goes further by uniquely mapping subtype-associated pathways, including T cell activation in TNBC (Fig. S8a), lymphocyte differentiation in ER+ tumors (Fig. 5e), and zinc ion responses in HER2+ profiles (Fig. S8b).

This global explanatory advantage substantiates the biological relevance of the features selected by DeepVaris. Accordingly, we further focus on the signatures exclusively identified by DeepVaris. To validate these unique signatures, we cross-reference them with established literature on BC proliferation, metastasis, and immune responses [34, 35]. Remarkably, >70% of the genes identified solely by DeepVaris have been previously reported in their respective subtypes (Fig. 5f; Supplementary Data III). Crucially, pathway enrichment analysis of these exclusive subsets confirms that they are not statistical noise, but rather related drivers in the hierarchical biological network, ranging from broad immune modulation [36, 37] to specific apoptosis regulation [38–40] (Figs 5g, S7d and e). This demonstrates that DeepVaris effectively rescues critical functional modules ignored by other methods.

Interestingly, within the TNBC cohort, DeepVaris uniquely identifies essential markers to T cell exhaustion (HAVCR2) and immune activation (CXCL9). These markers are expected to exhibit differential expression between pre- and on-treatment groups, particularly along the trajectory ended with exhausted CD8+ T cells (Tex lineage). Specifically, consistent with chronic immune exhaustion induced by prolonged tumor antigen exposure [41], HAVCR2 exhibits elevated expression in pretreatment samples and progressively increases along the Tex trajectory (Fig. 5h). Conversely, CXCL9, a chemokine vital for immune cell recruitment and a prognostic indicator for TNBC [42], shows lower baseline expression in pretreatment exhausted states (Fig. 5h). These findings highlight the capability of DeepVaris to capture potential temporal markers underlying subtle yet profound immune dynamics.

Finally, we study the relationship between features selected by DeepVaris and DEGs in each subtype. Unlike the previous example, DeepVaris recovers a relatively low proportion of DEGs (6%–28%; Fig. S7f). It is well known that a gene may be identified as important in DEGA due to its strong correlation with another gene that directly contributes to the phenotype. Consistent with this fact, most of the DEGs ignored by DeepVaris are strongly correlated with at least one of the DEGs selected by DeepVaris (Fig. S7g, **Methods**). Actually, this highlights a limitation of methods using marginal effects to evaluate variable importance. Differently, DeepVaris, a regression-based framework, avoids this redundancy. Indeed, we select many important genes, which are not DEGs but closely related to BC treatment as shown in pathway enrichment analysis (Fig. 5g: genes in red). For example, CTLA4 has been reported to express significantly higher in TNBC samples and influence the prognosis of TNBC patients through leukocyte differentiation and T cell activation pathways [43], and NKG7 is required for intratumor T-cell accumulation and antitumor immunity [44]. By moving beyond marginal associations to capture cooperative biological networks, DeepVaris provides a deeper interpretation of complex phenotypes.

## Discussion

Identifying critical biomarkers to diseases remains a core challenge in biology. Here, we introduce DeepVaris, a deep learning framework that frames feature selection as explaining a pretrained CNN predictor through surrogate modeling. Extensive simulations and real-world examples demonstrate its advantages over existing methods. Crucially, our findings highlight a key methodological insight that methods producing similar predictive accuracy often select different features. This divergence can substantially affect their downstream biological interpretation.

Recent DNN-based methods, such as SurvNet and DeepLink, handle nonlinearities by extracting FIS from trained networks. To control FDR, they typically introduce artificial variables and apply prespecified statistical thresholds (e.g. FDR $\leqslant 5\%$). In our examples, they may identify fewer important features even enlarging the FDR threshold, suggesting their underlying shortcomings. Differently, DeepVaris works well in these examples by leveraging the robust feature-extraction capability of CNNs and enforcing feature consistency across batches during surrogate modeling. This strategy rescues complete, synergistic biological modules. Furthermore, DeepVaris learns feature importance from a stable, pretrained CNN. This avoids the biased FIS estimations that unstable DNN models often produce in other methods.

To ensure that DeepVaris captures true synergistic signals rather than noise or artifacts introduced by the input representation, we conduct systematic permutation and architecture analyses (Fig. S9). First, we verify that the performance of DeepVaris is independent of the tabular data arrangement (i.e. CNN spatial inductive bias). Specifically, we randomly permute the order of the input features (Shuffled *X*). Shuffling the features inevitably alters the local receptive fields of the CNN. However, DeepVaris trained on feature-shuffled data has comparable performance with the original one (Fig. S9). These results indicate that DeepVaris does not rely on any arbitrary spatial arrangement.

Second, to explicitly demonstrate that DeepVaris effectively bounds the false positive rate and ignores pure noise, we randomly permute the phenotype labels (Shuffled *Y*). Under this null condition, the model fails to establish any valid tipping point in the *λ*-scanning process (Fig. S9a). Consequently, predictive accuracy persistently collapses to random chance (*∼*0.5) across all datasets (Fig. S9b). These results suggest that DeepVaris does not blindly accumulate noise to inflate performance.

Third, to further examine whether the selected variables are mainly driven by the CNN architecture or by discretization itself, we introduce two MLP-based selection baselines: raw MLP, which learns variable importance directly from the original continuous variables, and Discretized MLP, which uses the same discretized input representation as DeepVaris but replaces the CNN predictor with an MLP (Fig. S9d, **Methods**). The comparison between DeepVaris and Discretized MLP evaluates the contribution of the CNN-based selection pipeline beyond discretization alone, whereas the comparison between Raw MLP and Discretized MLP provides an empirical assessment of the information loss introduced by discretization under the same nonspatial architecture. Both MLP variants show inferior feature selection performance compared with DeepVaris, giving either higher FDR or lower TR (Fig. S9d). These results suggest that the performance of DeepVaris cannot be explained by discretization alone, and that replacing the CNN predictor with a nonspatial MLP does not achieve comparable feature selection performance. Together, these complementary analyses support the methodological robustness and soundness of DeepVaris.

Despite these advantages, certain limitations remain. DeepVaris currently requires more computational time to train IM-Net than other methods like SurvNet. We aim to optimize its efficiency in future work. Additionally, DeepVaris relies heavily on the quality of the pretrained CNN. Training a robust CNN is generally straightforward. However, challenges arise in ultra-high-dimensional, low-sample-size scenarios where variables far outnumber samples (*p ≫ n*). For such cases, we propose a two-stage hybrid strategy. First, we use computationally lighter methods (e.g. SurvNet) with a relaxed FDR threshold (e.g. 70%) as a coarse noise filter. Next, we apply DeepVaris to purify the feature space. We validate this strategy through simulations. DeepVaris successfully reduces the FDR by over 40% while maintaining a comparable true positive rate (Fig. S10). This confirms its ability to effectively purify spurious variables. Still, the initial filtering step might inadvertently discard some important signals. Therefore, fundamentally increasing the sample size remains the most robust solution for complex biological datasets.

## Methods

### DeepVaris

**Overview.** Let $D=\{(\mathbf{X}_{i} \in \mathbb{R}^{p}, y_{i})\}_{i=1}^{n}$ represent a dataset of *n* observations. Here, $\mathbf{X}_{i}=(x_{i1},\cdots ,x_{ip})$ denotes *p* features for the *i*th sample, and $y_{i}$ represents its corresponding phenotype. DeepVaris is an explainable deep learning framework. It selects features contributed to the phenotype through three sequential steps: (i) data discretization, (ii) CNN predictor construction, and (iii) feature importance learning via surrogate modeling.

**Step 1: data discretization.** To leverage the strengths of CNN, we first convert tabular data into an image format. We discretize continuous variables using one-hot encoding. For instance, consider feature $x_{i1}$. We assign $z_{i1}=k-1$ if $q_{k-1} < x_{i1} \leqslant q_{k}$, where $\{q_{1}, \cdots , q_{k}\}$ are predefined quantiles. Consequently, the input vector $\mathbf{X}_{i}$ transforms into a *k × p* binary matrix $\mathbf{Z}_{i}$. This matrix serves as an image-like input. Throughout this paper, we set *k=10* (Fig. S11a-b).

**Step 2: CNN predictor construction.** Next, we construct a CNN predictor, denoted as $\mathcal{P}$. It maps the image input $\mathbf{Z}_{i}$ to the phenotype $y_{i}$. The architecture includes one convolutional layer and two fully connected layers (FCLs) with ReLU activations. The convolutional layer applies 32 kernels of size *10 × 3*. The first FCL contains 64 neurons. The second FCL matches the specific task. It outputs a single continuous value for regression or a probability vector via softmax for classification. To prevent overfitting, we apply $L_{1}$ regularization.

**Step 3: feature importance learning.** Finally, we interpret the predictor $\mathcal{P}$ to identify key variables using surrogate modeling. Specifically, we employ Importance Mapping Net to learn a masked input, $\hat{\mathbf{Z}}_{i}$, in order to mimic the predictive behavior of the pretrained CNN, ensuring $\mathcal{P}(\hat{\mathbf{Z}}_{i}) \approx y_{i}$. The output of Importance Mapping Net is transformed by a sigmoid activation to obtain the sample-specific continuous importance score matrix $\mathbf{F}_{i} \in [0, 1]^{p}$.

**Architecture of Importance Mapping Net.** This network utilizes residual blocks and FCLs (Fig. 1b). The first layer is a 2D convolution. It extracts local features using one input channel, 32 output channels, and a *3 × 3* kernel. Subsequent layers form 10 residual blocks to capture deep representations. Each block contains four convolutional layers with matched padding and dilation settings. We apply Batch Normalization and ReLU activations after each layer.

**Variable importance.** We define $B_{i,j}=I(F_{i,j}\geq 0.5)$ as an indicator of whether the *j*th variable is important to the *i*th sample, and thus $\mathbf{B}_{i}\in \mathbb{R}^{p}$ indicates the importance of each variable to the *i*th sample. Because the indicator function is non-differentiable, we use a straight-through estimator during backpropagation. Specifically,


\begin{align*} & \widetilde{B}_{i,j}=F_{i,j}+\left(I(F_{i,j}\geq 0.5)-F_{i,j}\right)_{\mathrm{detach}}, \end{align*}

where $(\cdot )_{\mathrm{detach}}$ denotes a stop-gradient operation: it retains the numerical value of its argument in the forward pass but is treated as a constant with zero gradient in the backward pass. Therefore, $\widetilde{B}_{i,j}$ equals the hard indicator $I(F_{i,j}\geq 0.5)$ in the forward pass, but has the gradient of $F_{i,j}$ in the backward pass.

Accordingly, the importance score of the *j*th variable is defined as


\begin{align*} & I_j=\frac{1}{n} \sum_{i=1}^n B_{i,j} \in [0,1]. \end{align*}

A value of $I_{j}=1$ indicates the feature is essential for all samples. The total number of selected features is


\begin{align*} &L_s = \sum_{j=1}^p I_j.\end{align*}

Similar to LASSO, we penalize model complexity by adding $L_{s}$ to the loss function.

Furthermore, true biological drivers should be consistently important across different data batches. To suppress false discoveries, we introduce a consistency loss $L_{c}$. It explicitly penalizes the variance of importance scores across batches:


\begin{align*} &L_c=\frac{2}{n(n-1)}\sum_{1\le i < k\le n}\|\mathbf{F}_i-\mathbf{F}_k\|_1+\frac{1}{n}\sum^{n}_{i=1}\sum^p_{j=1}\left(F_{i,j}-\frac{1}{n}\sum^n_{m=1}F_{m,j}\right)^2,\end{align*}

where $\|\mathbf{a}\|_{1}=\sum _{i} |a_{i}|$ for any vector $\mathbf{a}$.

**Loss function of DeepVaris.** The loss of DeepVaris is given by $L_{p}+\lambda L_{s}+L_{c}$, where $L_{p}$ is the prediction loss of the surrogate model given below, and *λ* controls the sparsity penalty. For continuous response,


\begin{align*} & L_p=\frac{1}{n}\sum^n_{i=1}(y_i-\hat{y}_i)^2, \end{align*}

where $\hat{y}_{i}$ is the predicted value based on surrogate input $\hat{\mathbf{Z}}_{i}$ and for binary or categorical response,


\begin{align*} & L_p=- \frac{1}{n} \sum_{i=1}^{n} \sum_{l=1}^{C} y_{i,l} \log(\hat{y}_{i,l}), \end{align*}

where $\hat{y}_{i,l}$ is the predicted value based on surrogate input $\hat{\mathbf{Z}}_{i}$, and *C* is the number of categories.

### Training of DeepVaris

To train our models, the dataset is divided into training set, validation set and testing set. We first train a CNN predictor, and then train the Importance Mapping Net taking the CNN predictor as input to select important variables, and finally use selected variables to train a DNN as the final predictor. Parameters in the our neural networks are all initialized using Kaiming Uniform Initialization, and updated by Adam optimizer using mini-batch with batch size 32. To avoid overfitting, $L_{1}$ regularization and early-stop is used for regression and classification task, respectively.

**Reducing the training noise.** Stochastic components in DNN training, such as random gradient, early-stop strategy, mini-batch sampling, and different training initializations, introduce additional randomness into the output of DeepVaris. To get rid of this randomness, for each candidate *λ*, we run DeepVaris *m* times starting from different random seeds. The final output is defined as the intersection of the *m* sets obtained from these runs.

**Tuning the architecture of Importance Mapping Net.** The overfitting of Importance Mapping Net will make it unable to distinguish important features from noisy ones. To avoid this, we tune its structure using a random phenotype control. Specifically, we first generate a random phenotype $Y^{*}$ with the same length of true phenotype *Y* from normal distribution *N(0,1)*. Then, we run the model *m* times with different random initializations and get *m* sets of importance variables. If their intersection is empty, the Importance Mapping Net is considered not overfitted. Otherwise, we should reduce the complexity of the architecture. In our experiments, *m=5* works well for simulations, and *m=10* for real data analysis. Note that this number may vary in different analysis.

**Choosing *λ*.** The hyperparameter *λ* controls feature sparsity. We monitor $L_{u,\lambda }$, the CNN prediction loss, when fed only the discarded variables. When *λ* is small, DeepVaris selects almost everything. The discarded set contains pure noise, yielding poor accuracy. As *λ* increases, DeepVaris inevitably drops important variables into the discarded set, causing a sudden performance spike in $L_{u,\lambda }$. We dynamically identify this tipping point to select the optimal *λ* (Fig. 1d). To facilitate the selection, we first use $L_{p}$ from our CNN predictor to estimate the scale of *λ* needed to make $\lambda L_{s}$ be comparable with $L_{p}$. In addition, we usually have some rough ideas on the number *s* of important variables, e.g. usually $10 \leq s \leq 1000$. Thus, we have a rough range for *λ*, say, $[\lambda _{min}, \lambda _{max}]$, typically, $[10^{-5}, 10^{-1}]$. Second, we take *K* different values from $[\lambda _{min}, \lambda _{max}]$, and get $L_{u,\lambda _{i}}$ for $\lambda _{i} (i=1,2,\cdots , K)$. Finally, we set *λ* to $\lambda _{i}$, if $L_{u,\lambda }$ has a sharp change at $\lambda _{i}$. If multiple tipping points occur, we choose the first major change point.

**Final predicting model.** While the CNN predictor enables feature selection, discretizing continuous variables incurs minor information loss. To maximize final accuracy, we build the ultimate predictor using a standard DNN taking input the original, undiscretized values of the selected features. This approach simplifies the architecture, improves computational efficiency, and captures full continuous information.

### Simulation

Let *s* be the number of true features, *p* be the total number of features, and *n* be the sample size. We denote the phenotype as *Y*, feature matrix as $\mathbf{X}=(x_{ij})_{n\times p}$, true features as $X_{j}, 1\le j\le s$, and noisy features as $X_{j}, s+1\le j\le p$. We simulate six examples to evaluate the performance of different methods across varying noise levels and nonlinear effects, on classification tasks (Datasets 1–3) and regression tasks (Datasets 4–6).

**Dataset 1.** We start with a simple classification task where variables are independent. Specifically, we simulate *n=10 000* samples, which are randomly divided into two classes, $C_{1}$ and $C_{2}$, with equal size. Variables are generated from uniform distribution on [0,1], i.e. $x_{ij}\sim U(0,1), 1\le j\le p$ where *p=784*, and true variables are set by


\begin{align*} &x_{ij}\leftarrow x_{ij}+(2\alpha_j-1)\delta_j, i\in C_1, 1\le j\le s,\end{align*}

where *s=64*, $\alpha _{j} \sim \mathrm{Bernoulli}(1/2), \delta _{j} \sim U(0.1,0.3)$.

**Dataset 2.** In this example, we simulate correlated variables by taking images of “0” in MNIST dataset as samples (*n=6903*) and image pixels as features (*p=784*). All samples are randomly divided into two classes, $C_{1}$ and $C_{2}$, with equal size, and true variables are generated by


\begin{align*} &x_{ij}\leftarrow x_{ij}+(2\alpha_j-1)\delta_j, i\in C_1, 1\le j\le s,\end{align*}

where *s=64*, $\alpha _{j} \sim \mathrm{Bernoulli}(1/2), \delta _{j} \sim U(0.1,0.3)$.

**Dataset 3.** We further simulate true variables with inflated variance to increase noise levels. The generation of $\mathbf{X}$ and the classification of samples follow the same procedure as in Dataset 1, with *n=10 000* and *p=784*. Differently, true variables are generated as


\begin{align*} &x_{ij}\leftarrow x_{ij}+(2\alpha_{ij}-1)\delta_{ij}, i\in C_1, 1\le j\le s,\end{align*}

where *s=64*, $\alpha _{ij} \sim \mathrm{Bernoulli}(1/2), \delta _{ij} \sim U(0.8,1)$.

**Dataset 4.** For regression tasks, we first simulate a dataset following nonlinear relationships with interactions between variables. Specifically, we simulate *n=10 000* samples and generate variables from uniform distribution on [−1,1], i.e. $x_{ij}\sim U(-1,1), 1\le j\le p$ where *p=784* and *s=64*. The phenotype is generated by


\begin{align*} Y &= \sum_{j=1}^{16} \beta_{j} X_{j} + \sum_{j=17}^{32} \beta_{j} \sin X_{j} + \sum_{j=33}^{48} \beta_{j} e^{X_{j}} + \sum_{j=49}^{64} \beta_{j} \max(0, X_{j}) \notag \\ &\quad + \beta^{\prime}_{1} X_{15} X_{16} +\beta^{\prime}_{2} X_{31} X_{32} + \beta^{\prime}_{3} X_{47} X_{48} + \beta^{\prime}_{4} X_{63} X_{64} + \varepsilon_{i}, \end{align*}

where $\beta _{j} = (2\alpha _{j} - 1) b_{j}, \alpha _{j} \sim \mathrm{Bernoulli}(1/2), b_{j} \sim U(1,3)$, $\beta ^{\prime}_{1}, \beta ^{\prime}_{2}, \beta ^{\prime}_{3}$ and $\beta _{4}^{\prime}$ follow the same distribution as $\beta _{j}$, and $\varepsilon _{i} \sim \mathcal{N}(0,1)$.

**Dataset 5.** To study the robustness of different methods against noise, we increase the variance of the error term, i.e. $\epsilon _{i}\sim \mathcal{N}(0,4)$, while keeping the remaining settings consistent with Dataset 4.

**Dataset 6.** To study the efficiency of different methods under stronger nonlinear relationships, we increase the weights of the nonlinear terms, i.e.


\begin{align*} Y &= \sum_{j=1}^{16} \beta_{j} X_{j} + 2\sum_{j=17}^{32} \beta_{j} \sin X_{j} + 2\sum_{j=33}^{48} \beta_{j} e^{X_{j}} + 2\sum_{j=49}^{64} \beta_{j} \max(0, X_{j}) \notag \\ &\quad + \beta^{\prime}_{1} X_{15} X_{16} +\beta^{\prime}_{2} X_{31} X_{32} + \beta^{\prime}_{3} X_{47} X_{48} + \beta^{\prime}_{4} X_{63} X_{64} + \varepsilon_{i}. \end{align*}

The rest remains the same as Dataset 4.

**MLP baseline.** To assess whether the CNN inductive bias drives variable selection, we compare DeepVaris with two MLP-based selection baselines. The first baseline, Raw MLP + Importance Mapping Net (IM-Net), learns variable importance directly from the original continuous variables. The second baseline, Discretized MLP + IM-Net, uses the same discretized input representation as DeepVaris but replaces the CNN predictor with an MLP. All methods are run with the same 10 random seeds on three representative simulation datasets (datasets 1, 3, 6).

### Competing methods

**Stabl** [7] is a penalized regression method that selects the tuning parameter based on the stability of variable selection, while controlling the FDR under regular assumptions, such as the linearity of the true model. Stabl is implemented in Python and available at https://github.com/gregbellan/Stabl.

**SurvNet** [15] is a DNN-based variable selection method that controls FDR by introducing surrogate variables and progressively eliminating the least relevant variables until a prespecified FDR threshold is reached. We tune the target FDR threshold, $\eta ^{*}$, as the primary parameter controlling the stringency of variable selection. Other settings follow the original paper. SurvNet is implemented in Python and available at https://github.com/zixuans/SurvNet.

**DeepLink** [16] is a DNN-based statistical inference framework based on knockoffs. It first constructs knockoff variables using an autoencoder and subsequently trains an MLP for feature selection with FDR control. For DeepLink, we primarily vary the target FDR level, *q*, while retaining the recommended settings from the original study for the autoencoder and MLP training procedure. DeepLink is implemented in Python and available at https://github.com/zifanzhu/DeepLINK.

**HiDe-MK** [17] is another DNN-based variable selection method that controls FDR within a knockoff framework. It was specifically designed to identify putative causal genetic variants, particularly low-frequency and rare variants. We get results under different target FDR levels while other settings follow the original paper. HiDe-MK is implemented in Python and available at https://github.com/Peyman-HK/De-randomized-HiDe-MK.

### Evaluation metrics

**Metrics for simulation.** (i) Initial (final) error. Let $y_{i}, i=1,2,...,n$ be the observed values and $\hat{y}_{i}$ be the predicted values based on all features (selected features), where *n* is the sample size. For regression tasks, $\mathrm{error}=\frac{1}{n}\sum ^{n}_{i=1}(y_{i}-\hat{y}_{i})^{2}$. For classification tasks, $\mathrm{error}=(1-\frac{1}{n}\sum ^{n}_{i=1}I(y_{i}=\hat{y}_{i}))*100$. (ii) False discovery rate (FDR). FDR is defined as the proportion of the noisy variables in selected variables. (iii) True ratio (TR). TR is defined as the ratio of selected true features to all true features.

**Metrics for real data analysis.** Denote True Positives as TP, False Positives as FP, True Negatives as TN, and False Negatives as FN. (1)


\begin{align*} &\mathrm{Accuracy} = \frac{\mathrm{TP+TN}}{\mathrm{TP+TN+FP+FN}}.\end{align*}

(2) AUC. Define


\begin{align*} &\mathrm{Sensitivity} = \frac{\mathrm{TP}}{\mathrm{TP} + \mathrm{FN}},\end{align*}

\begin{align*} &\mathrm{Specificity} = \frac{\mathrm{TN}}{\mathrm{TN} + \mathrm{FP}},\end{align*}

and the ROC curve plots Sensitivity against (1 − Specificity) across various decision thresholds, while AUC quantifies the area under the ROC curve.

### Real data analysis

**Dream challenge dataset ($\boldsymbol{p=9193, n=1569}$).** The DREAM challenge dataset collected nine publicly available vaginal microbiome data from labor pregnancies [45]. It aims to classify preterm and term pregnancies using microbiome dataset, including 5468 phylotypic variables and 3725 taxonomic variables. The data contain 1569 samples from 580 individuals, including 336 individuals delivered at term and 244 individuals delivered preterm.

**scRNA mouse dataset ($\boldsymbol{p=1046, n=5282}$).** Chen *et al*. [46] performed analysis of the adult mouse hypothalamus and identified different cell types based on clustering. Following [15], we take 5282 cells from two non-neuronal cell types, OPC and MO, which demonstrate distinct stages of oligodendrocyte maturation. A total of 1046 genes are used as features for classification.

For cell clustering analysis, we first create Seurat object with count matrix containing only selected genes (CreateSeuratObject function in R package *Seurat*, v5.1.0). After data normalization and scaling, cells are visualized using tSNE. We evaluate the clustering quality of each cell type using SC (R package cluster, v2.1.6). The SC for cell *i* is defined as $S(i)=\frac{b(i)-a(i)}{max(a(i),b(i))}$, where *a(i)* is the average distance between cell *i* and all the other cells in the same cell type, and *b(i)* is the minimum average distance between cell *i* and all the cells in any other cell type, and the distance is calculated based on the tSNE reduced data. In this way, SC quantifies how similar a cell is to its own cluster compared with other clusters, and thus assesses the clustering quality of cells. SC values range from −1 to 1, where higher values indicate better-defined clusters. Pathway enrichment analysis is performed using enrichGO function in R package *clusterProfiler* (v4.14.4). For differential expression analysis, DEGs are first identified using FindMarkers function in R package *Seurat* (v5.1.0). The threshold is calculated as the average fold change plus two standard deviations of the fold change. DEGs are then filtered by selecting markers with a fold change greater than the threshold (up-regulated) or smaller than the negative threshold (down-regulated).

**Breast cancer dataset ($\boldsymbol{p=6649, n=20\,186}$).** Bassez *et al*. [33] obtained scRNA-seq data of CD8+ T cells from patients with nonmetastatic, treatment-naive primary invasive breast carcinoma, treated with a single dose of pembrolizumab (Keytruda or anti-PD1) *∼*9*±*2 days prior to surgery. The samples were paired, consisting of a tumor biopsy taken immediately before anti-PD1 treatment (pretreatment) and another collected during surgery (on-treatment).

Pathway enrichment analysis is performed using enrichGO function in R package *clusterProfiler* (v4.14.4). The R package *slingshot* (v2.14.0) is used to infer pseudotime trajectories in T cells. The labels are predicted using k-means clustering method (kmeans function in R package stats, v4.1.1). Then, we utilize getLineages function in slingshot to fit a minimum spanning tree to identify lineage. In accordance with established biological knowledge, we set the root for CD8+ T cell dataset as CD8_Naive and the ends as CD8_Emra, CD8_Ex, and CD8_Rm. For DEGA, DEGs are first identified using FindMarkers function in R package *Seurat* (v5.1.0). Only significant genes (adjusted *P*-value*⩽*.05) with a fold change greater than the threshold (the average fold change plus two standard deviations) or smaller than the negative threshold are used. To study the relationship between genes identified by DeepVaris and DEGs from DEGA, we denote S1 as DEGs identified by DeepVaris, and S2 as DEGs ignored by DeepVaris. Then, for each gene *g* in S2, we count the number of genes in S1 strongly correlated with *g* (Pearson’s correlation test *P*-value*⩽*.05).

**Key Points**

- DeepVaris is a novel explainable AI framework that first trains a highly accurate CNN, and then uses surrogate modeling to decode it, precisely pinpointing features related to the phenotype.
- Previous methods rely on prespecified FDR thresholds, which may mistakenly discard real signals. Differently, DeepVaris combines sparsity regularization, feature consistency, and data-driven parameter selection to identify stable features and filter out noisy features.
- When handling extremely sparse and noisy data (such as microbiomes), DeepVaris successfully identifies valuable features. It not only finds strong signals but also rescues hidden disease networks and gene switches that seem weak on their own but become highly predictive when combined.
- DeepVaris proves its strong adaptability across real-world tasks, including predicting preterm birth, tracking cell maturation in neurodegenerative diseases, and analyzing immune exhaustion in breast cancer subtypes, where it extracts highly specific targets with direct potential for clinical treatment.

## Supplementary Material

Supplementary_material_bbag476

## Data Availability

Data for Dream challenge is available at https://github.com/gregbellan/Stabl/tree/main/Sample∖%20Data. The scRNA mouse dataset is available at the GEO repository https://www.ncbi.nlm.nih.gov/geo/query/acc.cgi?acc=GSE87544. Data for breast cancer is available at http://biokey.lambrechtslab.org. The code for DeepVaris, analysis and plots in this paper can be obtained at https://github.com/zlyx26/DeepVaris.
