# Supplementary material for: Interpretable deep neural network identifies robust biomarkers for diseases with mechanistic insights from omics data
Source: Brief Bioinform. 2026 Sep 7;27(5):bbag476. doi: 10.1093/bib/bbag476 (PMC13548326; doi:10.1093/bib/bbag476)
Supplement: Supplementary file 1 [file supplementary_material_bbag476.zip › supp_bbag476.pdf]

Supplementary material to
Interpretable deep neural network identifies robust biomarkers for diseases with mechanistic insights from omics data

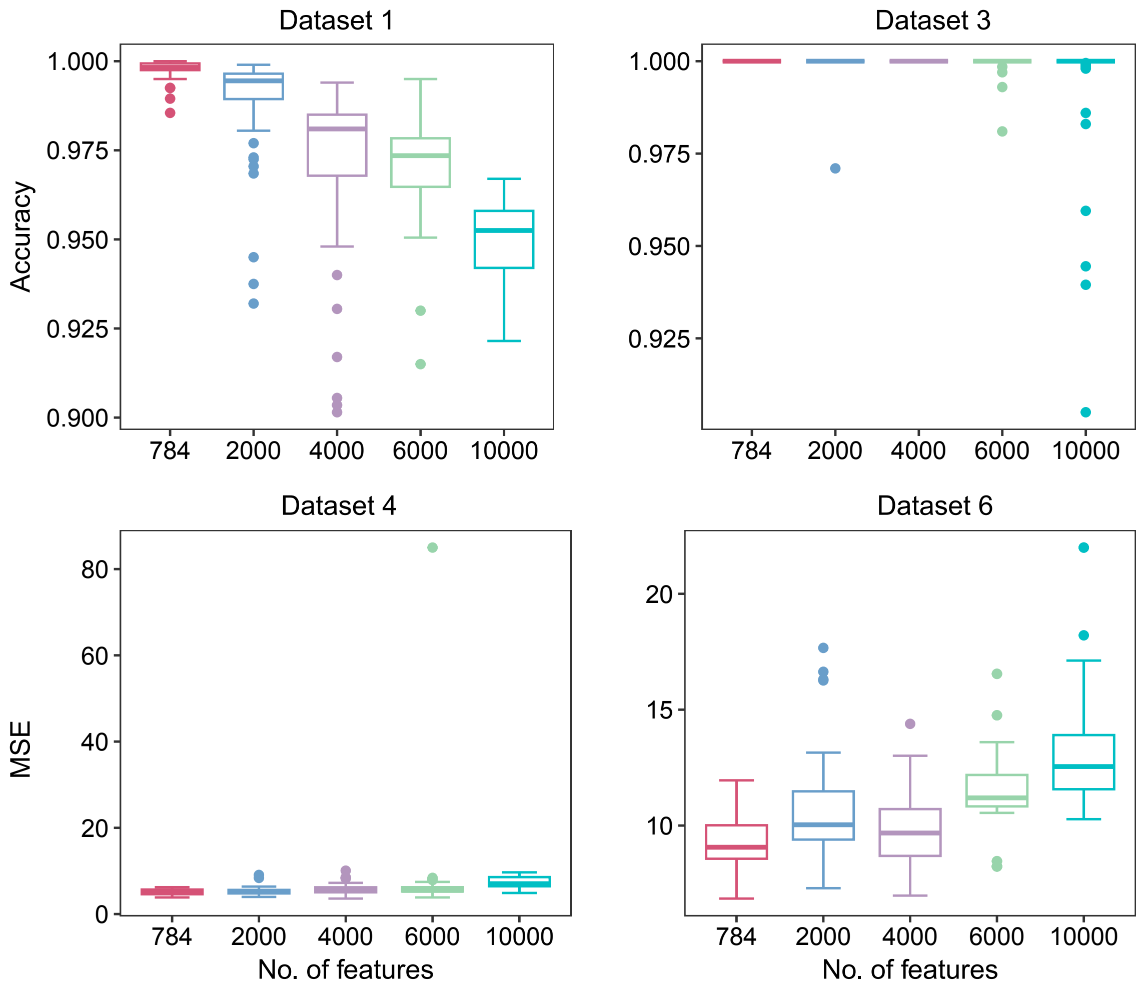

Figure S1: **The impact of the number of noisy variables on the performance of CNN.** We evaluate the performance of CNN on Dataset 1, 3, 4, and 6 with different numbers of noisy variables. We observe that CNN performs well in different cases.

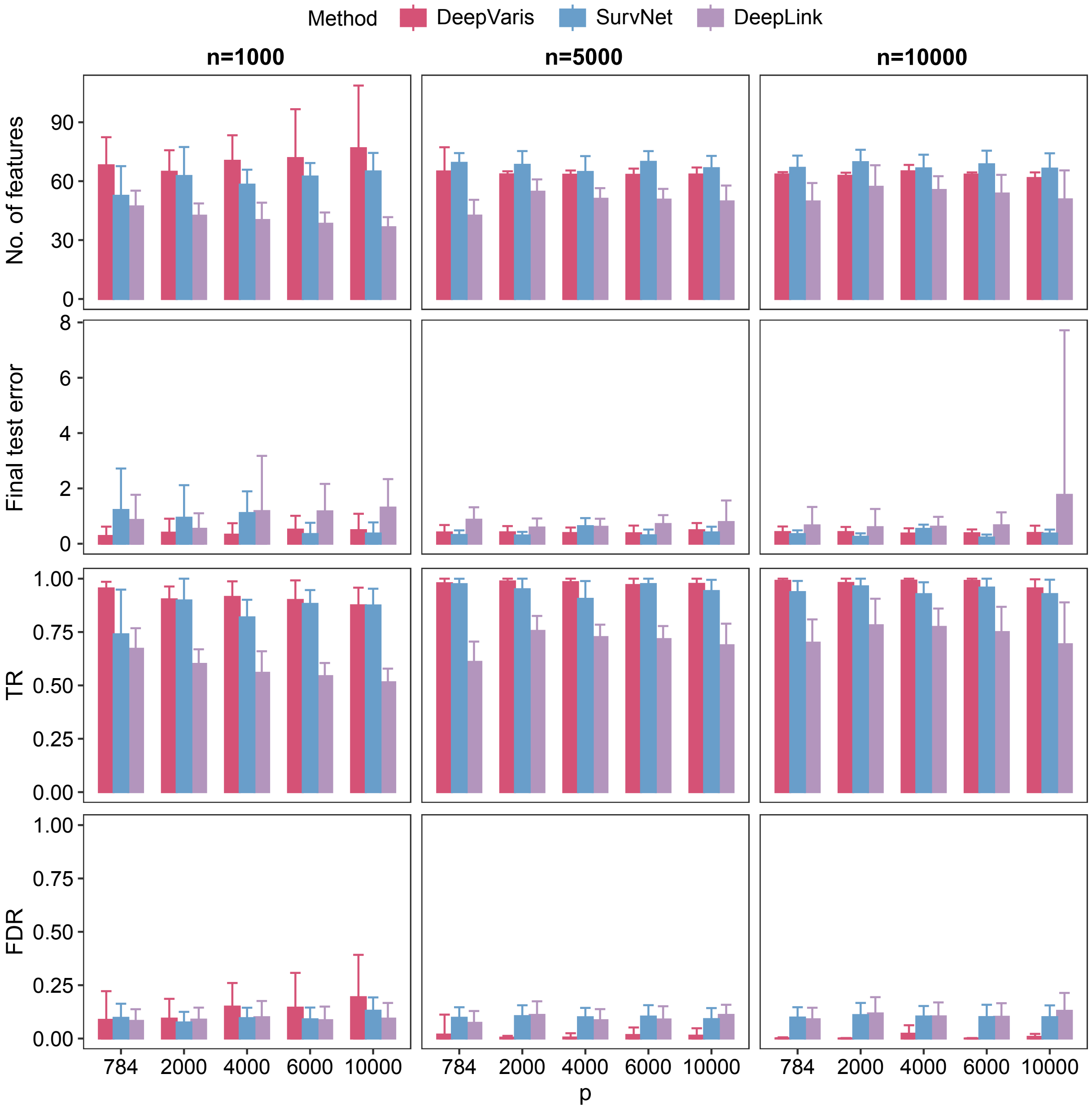

Figure S2: **Additional simulation results for Dataset 1.** We evaluate the performance of DeepVaris, Survnet and DeepLink across different sample sizes ($n = 1000, 5000, 10000$) and feature dimensions ($p = 784, 2000, 4000, 6000, 10000$).

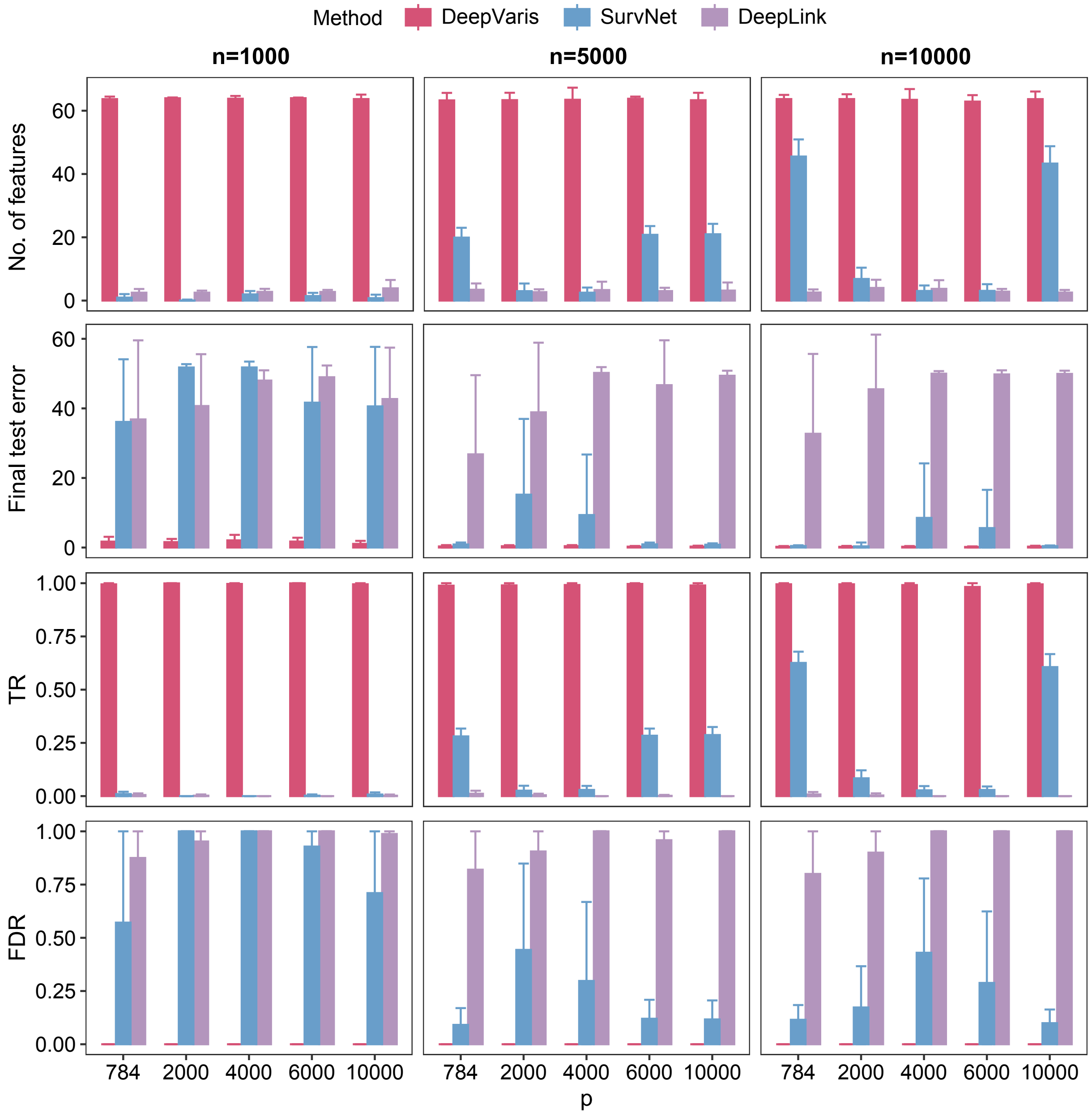

Figure S3: **Additional simulation results for Dataset 3.** We evaluate the performance of DeepVaris, Survnet and DeepLink across different sample sizes ($n = 1000, 5000, 10000$) and feature dimensions ($p = 784, 2000, 4000, 6000, 10000$).

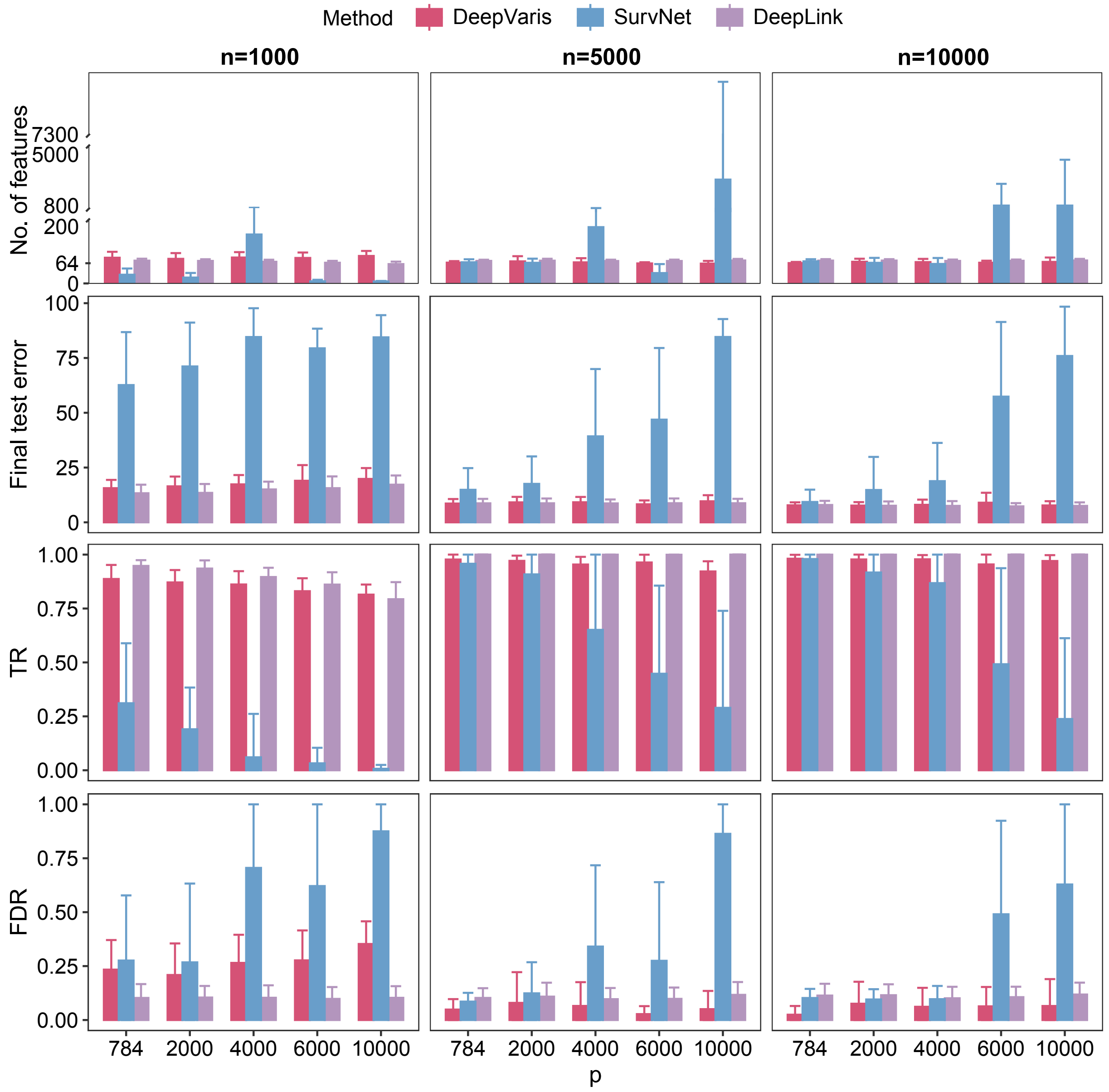

Figure S4: **Additional simulation results for Dataset 4.** We evaluate the performance of DeepVaris, Survnet and DeepLink across different sample sizes ($n = 1000, 5000, 10000$) and feature dimensions ($p = 784, 2000, 4000, 6000, 10000$).

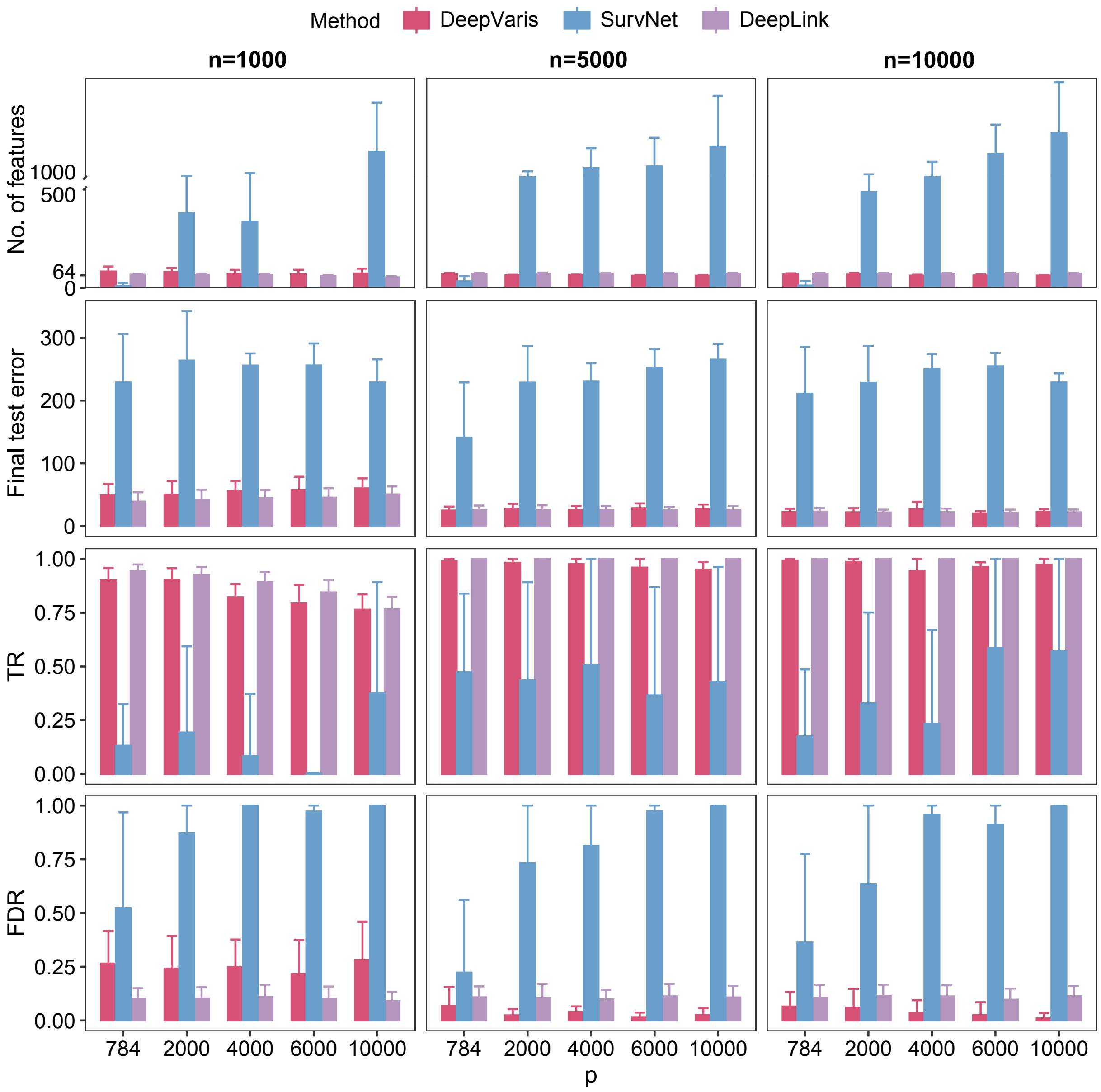

Figure S5: **Additional simulation results for Dataset 6.** We evaluate the performance of DeepVaris, Survnet and DeepLink across different sample sizes ($n = 1000, 5000, 10000$) and feature dimensions ($p = 784, 2000, 4000, 6000, 10000$).

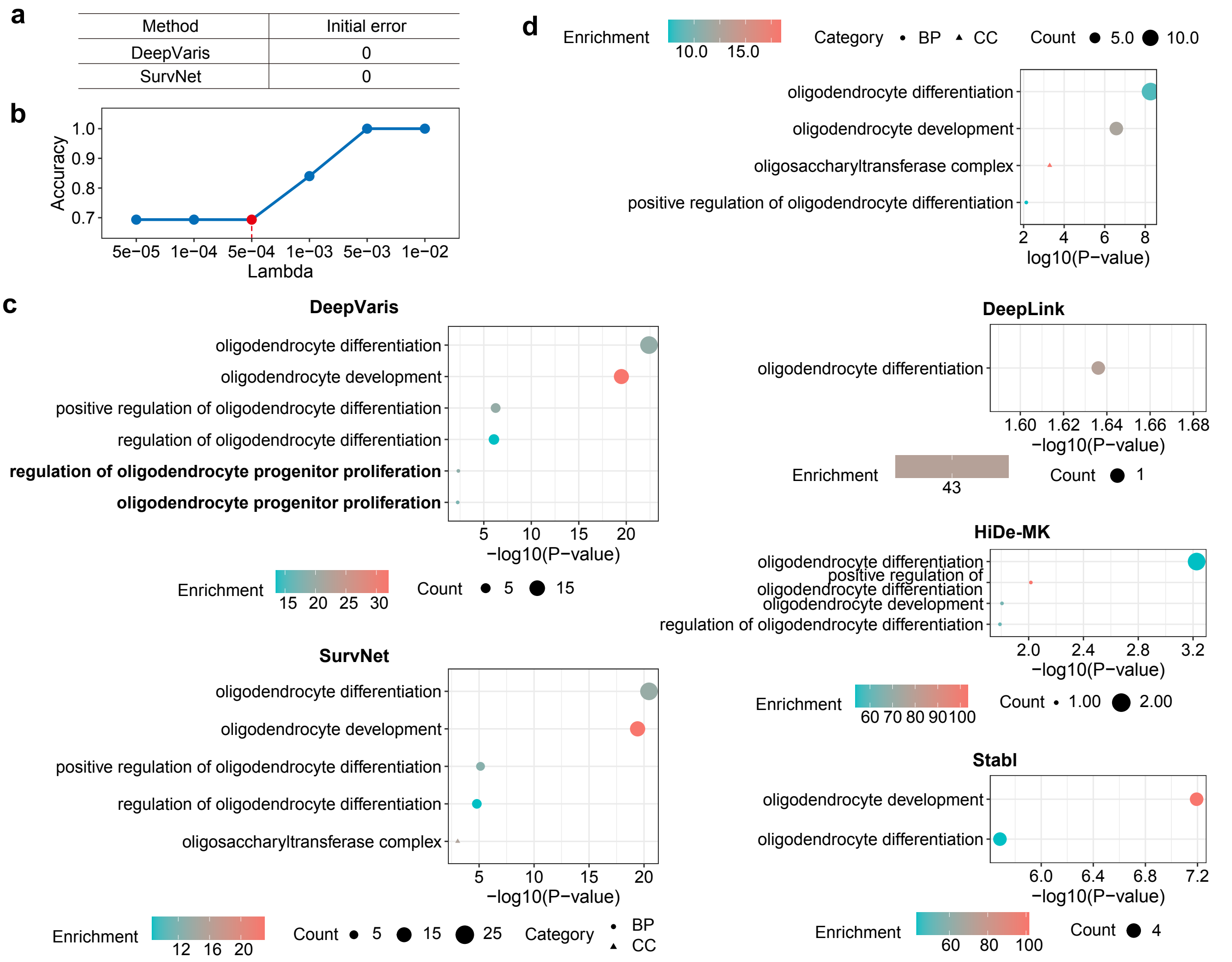

Figure S6: **Additional results for mouse hypothalamus dataset.** **a,** The initial errors of DeepVaris and SurvNet. **b,** The parameter λ in DeepVaris is chosen as the tipping point of test accuracy. **c,** The pathways related to oligodendrocyte maturation, enriched by genes selected by each method. **d,** The pathways related to oligodendrocyte maturation, enriched by non-DEGs selected by SurvNet.

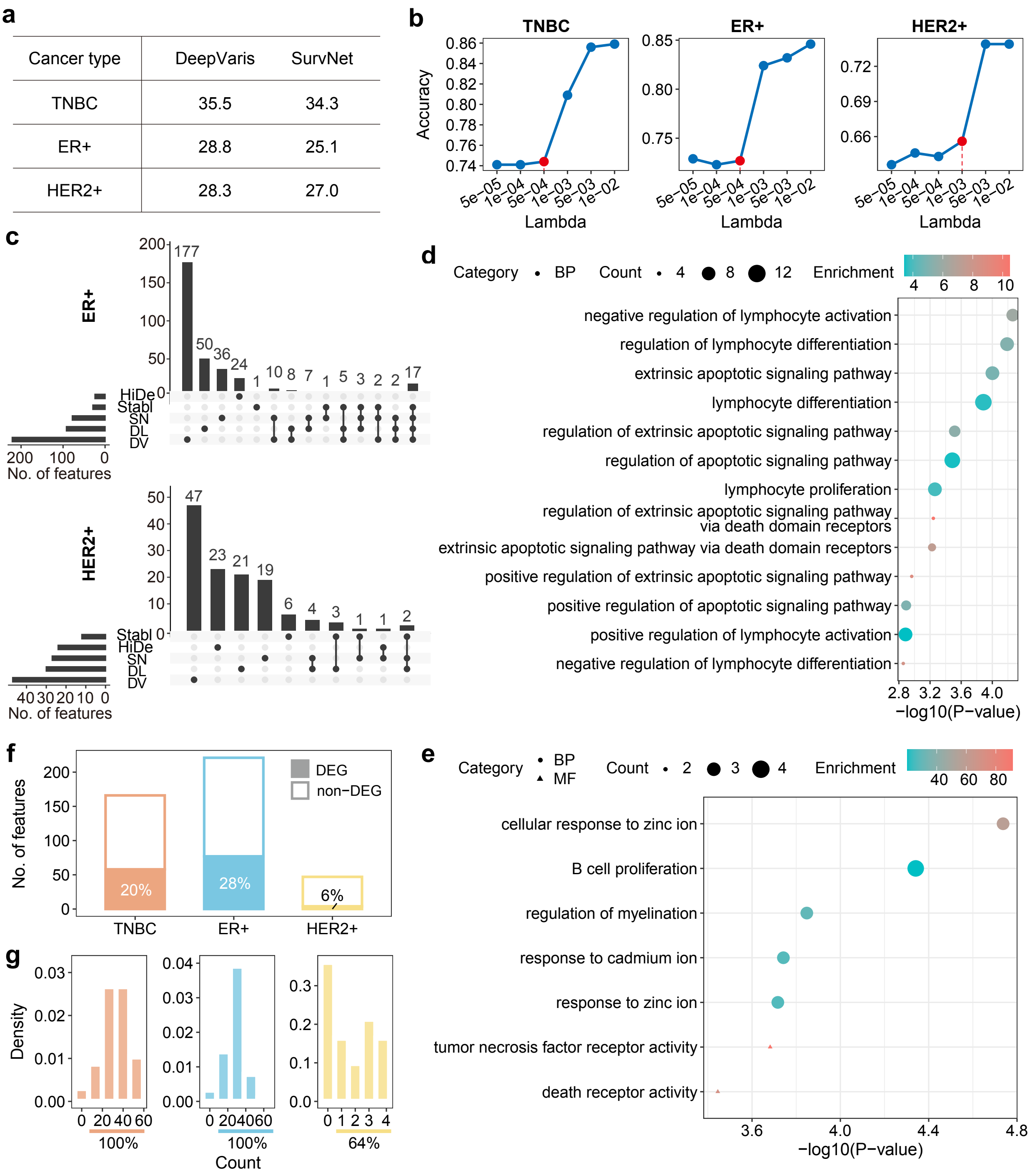

Figure S7: **Additional results for breast cancer treatment.** **a,** The initial errors of DeepVaris and SurvNet for each subtype of BC. **b,** The parameter λ in DeepVaris is chosen as the tipping point of test accuracy. **c,** The relationship between variables selected by different methods in ER+ and HER2+ BC data. **d-e,** The pathways enriched by genes exclusively selected by DeepVaris in **d,** ER+ and **e,** HER2+ data. **f,** The numbers of DEGs and non-DEGs in features seleted by DeepVaris. The percentages indicate the proportions of DEGs recovered by DeepVaris out of the total DEGs in each subtype. **g,** We perform a correlation test between DEGs identified by DeepVaris (denoted as S1) and those not identified (denoted as S2). For each gene g in S2, we count the number of genes in S1 strongly correlated with g (Pearson's correlation test p-value ¡ 0.05). The histogram illustrates the distribution of these counts, and the percentages represent the proportions of genes in S2 correlated with at least one of the DEGs selected by DeepVaris.

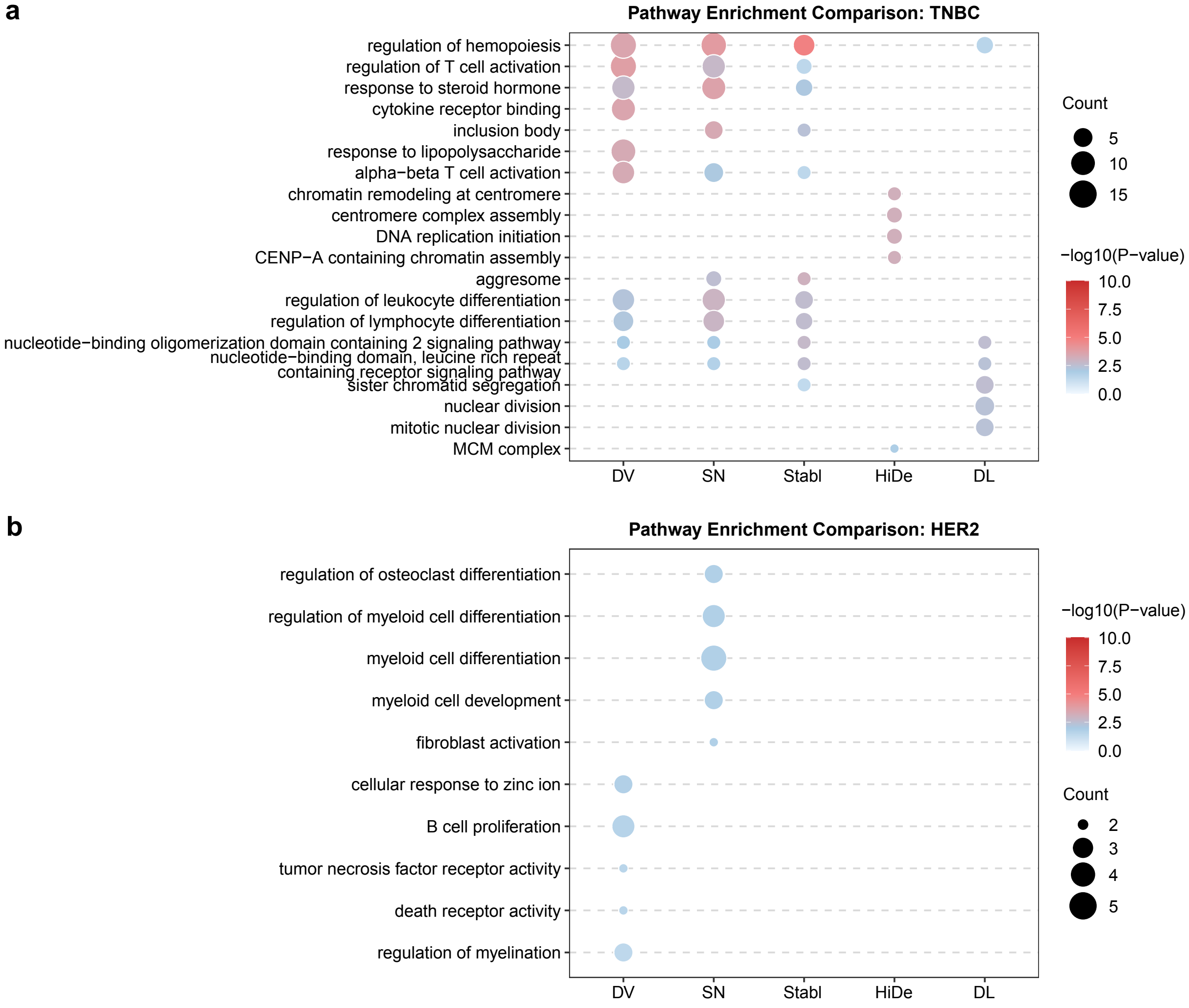

Figure S8: **Additional pathway enrichment comparison across breast cancer subtypes. a-b,** The top 5 most significantly enriched pathways derived from the optimal feature sets selected by DeepVaris and competing methods within the **a,** TNBC and **b,** HER2+ cohorts.

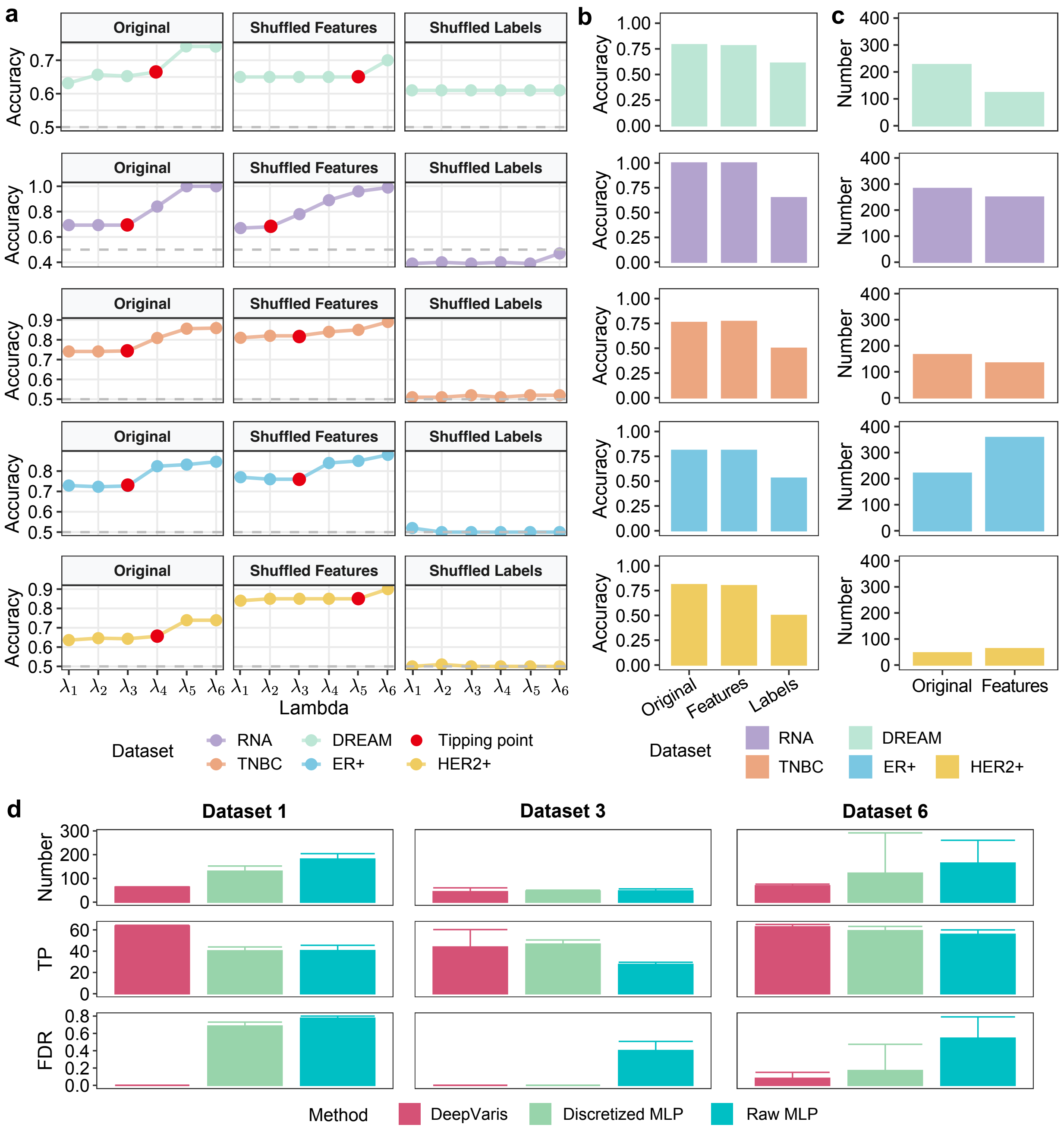

Figure S9: **DeepVaris demonstrates robustness via a-c, permutation tests and d, MLP baselines. a,** The λ is chosen based on the tipping point of test accuracy across the original data, feature-permuted data (Shuffled X), and label-permuted data (Shuffled Y). Both the original and Shuffled X configurations exhibit clear tipping points for feature selection, where the selected λ is colored in red, while the Shuffled Y null model completely fails to establish valid thresholds. **b,** Comparison of final predictive accuracy across different conditions. **c,** The number of selected features for the original and Shuffled X models. **d,** The performance of DeepVaris against two MLP baselines on dataset 1, 3 and 6: Raw MLP, which learns variable importance directly from the original continuous variables, and Discretized MLP, which uses the same discretized input representation as DeepVaris but replaces the CNN predictor with an MLP.

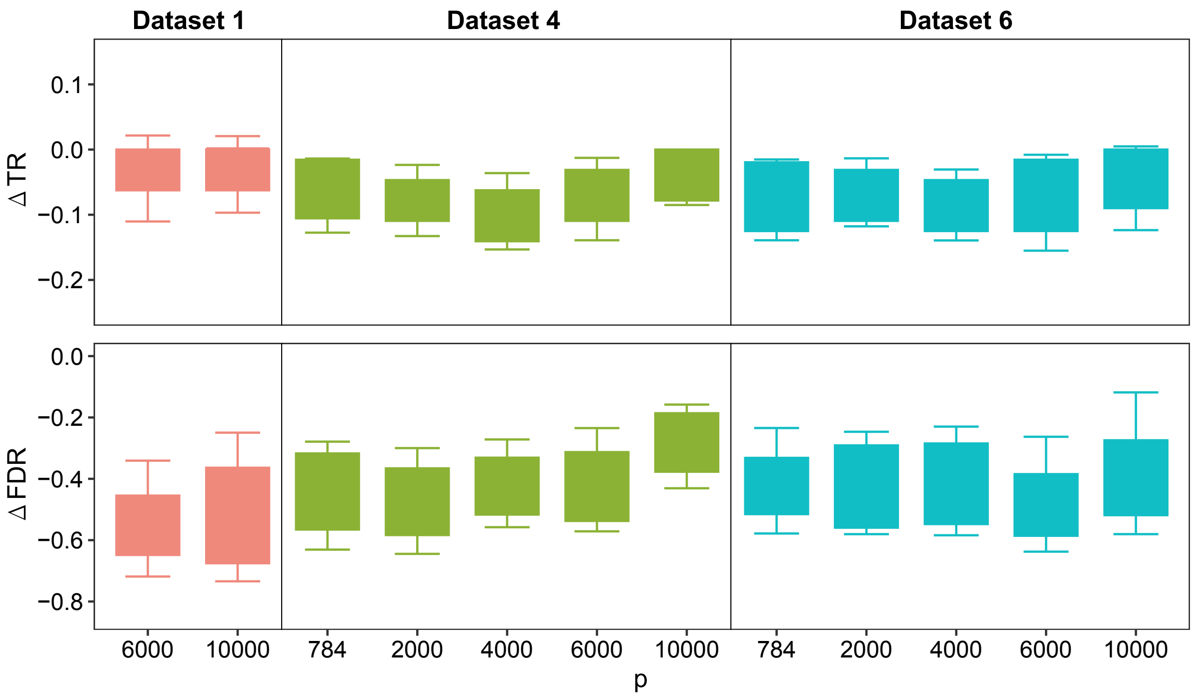

Figure S10: **Model improvement by applying DeepVaris on pre-selected features from other methods.** To evaluate the improvement in performance, we first apply other methods (Survnet for classification tasks and DeepLink for regression tasks) for variable selection by controlling the FDR under 70%, and subsequently use DeepVaris on these selected features. The difference in performance is defined as ΔFDR=FDR(DeepVaris)-FDR(other methods), and similarly for ΔTR. We observe comparable TR and significantly lower FDR.

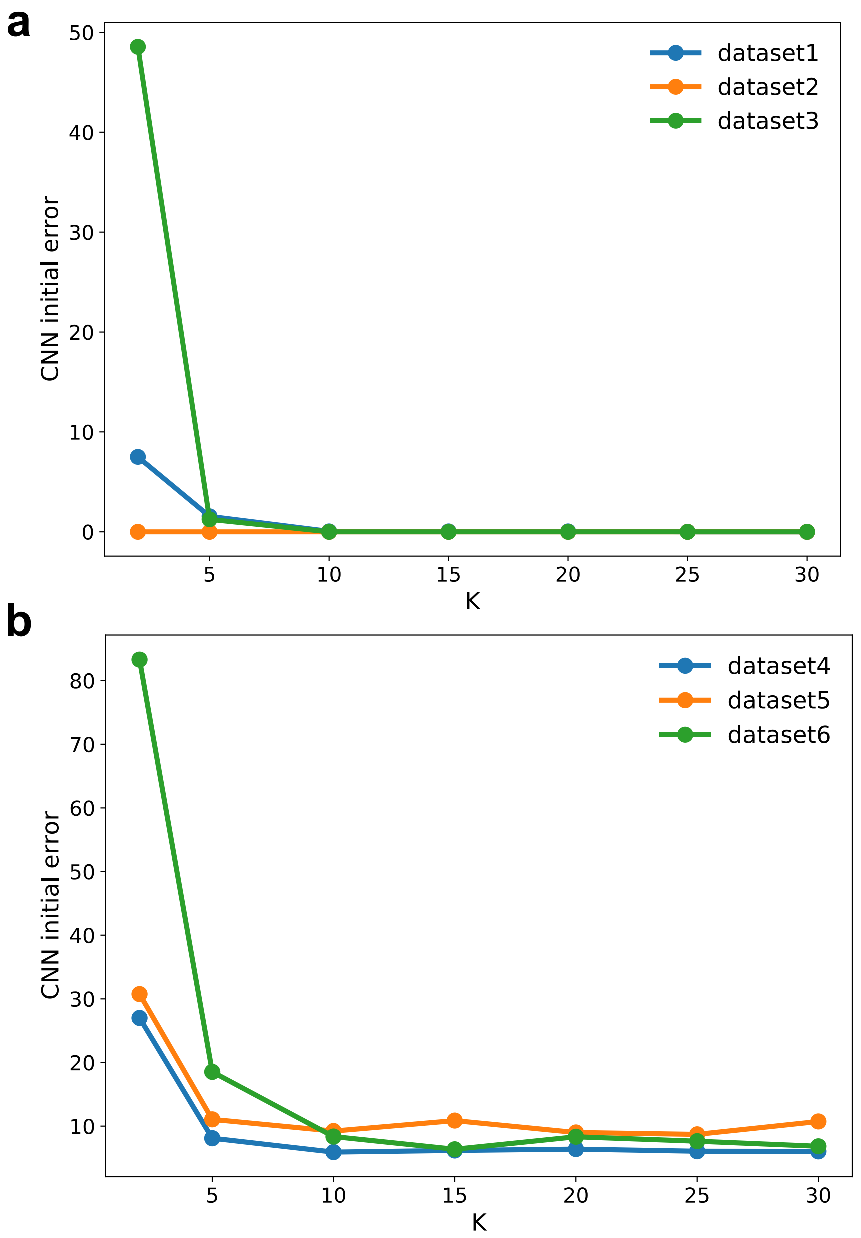

Figure S11: **Performance of CNN with different K on classification tasks (a) and regression tasks (b).** We can see that CNN performs well when $k = 10$.
